# Iranian Medicinal Plants: From Ethnomedicine to Actual Studies

**DOI:** 10.3390/medicina56030097

**Published:** 2020-02-26

**Authors:** Piergiacomo Buso, Stefano Manfredini, Hamid Reza Ahmadi-Ashtiani, Sabrina Sciabica, Raissa Buzzi, Silvia Vertuani, Anna Baldisserotto

**Affiliations:** 1 Department of Life Sciences and Biotechnology, Master Course in Cosmetic Sciences, University of Ferrara, Via Luigi Borsari 43, 44121 Ferrara, Italy; piergiacomo.buso@student.unife.it (P.B.); smanfred@unife.it (S.M.); sabrina.sciabica@student.unife.it (S.S.); raissa.buzzi@unife.it (R.B.); anna.baldisserotto@unife.it (A.B.); 2 Department of Basic Sciences, Faculty of Pharmacy, Tehran Medical Sciences, Islamic Azad University, Tehran 194193311, Iran; ahmadi@iaups.ac.ir; 3 Cosmetic, Hygienic and Detergent Sciences and Technology Research Center, Tehran Medical Sciences, Islamic Azad University, Tehran 194193311, Iran; 4 Ambrosialab S.r.l. University of Ferrara Spinof Company, Via Mortara 171, 44121 Ferrara, Italy

**Keywords:** Iranian traditional medicine, biological activities, pharmaceutical, cosmeceutical, nutriceutical

## Abstract

Iran has a rich and diverse cultural heritage, consisting of a complex traditional medicine deeply rooted in the history of the territory that goes back to the Assyrian and Babylonian civilizations. The ethnomedical practices that can be identifiable nowadays derive from the experience of local people who have developed remedies against a wide range of diseases handing down the knowledge from generation to generation over the millennia. Traditional medicine practices represent an important source of inspiration in the process of the development of new drugs and therapeutic strategies. In this context, it is useful to determine the state of the art of ethnomedical studies, concerning the Iranian territory, and of scientific studies on plants used in traditional Iranian medicine. Data regarding 245 plants used in Iranian ethnomedical practices and scientific studies conducted on 89 plants collected in the Iranian territory have been reported. All of the scientific studies here reported draw inspiration from traditional medicine. The World Health Organization (WHO) has repeatedly called for an intensification of the scientific validation processes of traditional medicines intended as an important contribution to public health in various parts of the world. The process of study and validation of Iranian ethnomedical practices appears to be at an early stage.

## 1. Introduction

Traditional medicine practices represent an important and often underestimated part of healthcare around the world. Moreover, traditional knowledge is a source of inspiration for researches on biological activities of vegetal extracts and pure compounds that can be obtained from them. A great number of lifesaving therapeutic assets belonging to modern medicine and new active compounds are derived from traditional knowledge and traditional uses of plants.

The awareness of this fact led to the drawing up of the World Health Assembly (WHA) resolution on Traditional Medicine (WHA62.13) and the WHO Traditional Medicine Strategy 2002–2005 and 2014–2023. These documents aim to integrate at the international level national healthcare systems with traditional knowledge and practices through an assessment of safety, efficacy, and quality of the treatments. In order to achieve these objectives, it is necessary to properly carry out scientific researches; the biological activities of the plants used must be tested, and the effectiveness of the treatments both “in vitro” and “in vivo” must be assessed considering the risk/benefit profile. Thus, one of the main raised issues—related to the use of traditional practices in national policies and regulations—is the lack of research data [1].

WHO defines traditional medicine as follows: “Traditional medicine has a long history. It is the sum total of the knowledge, skill, and practices based on the theories, beliefs, and experiences indigenous to different cultures, whether explicable or not, used in the maintenance of health as well as in the prevention, diagnosis, improvement or treatment of physical and mental illness” [2].

In light of a literature search, the traditional Iranian medicine (also known as Persian medicine) results, particularly rich in information, which can justify new studies regarding the therapeutic use of plants and vegetal extracts; it consists of the totality of the knowledge passed down through the generations and of the practices based entirely on observations and practical experience used, from ancient times to nowadays, in diagnosis, prevention, and elimination of diseases in the Iranian territory [3].

In this context, it was of great interest for us to collect scientific reports/studies, deriving from traditional practices, regarding health properties: biological activities of native Iranian plants proper to the medicinal, dermo-cosmetic, and nutriceutical use, in order to provide a complete overview of the scientific knowledge and establish a starting point for further research. Particular attention was paid to works that open up research possibilities on new therapeutic assets that deserve a follow-up to determine the efficacy of the reported biological activities in vivo.

## 2. Materials and Methods

### The Present Review Was Performed Adopting The Following Databases: Scifinder, Pubmed, Google Scholar

Selection criteria were defined, including articles regarding ethnobotanical studies on medicinal plants traditionally used in the Iranian territory and articles reporting scientific studies on plants grown and collected in Iran, including biological activities that can be spent in the pharmaceutical, cosmetic/cosmeceutical, nutraceutical fields. Particular attention was paid to works that may open up research paths to new therapeutic assets. All the studies reported in this review draw inspiration from Iranian traditional medicine practices.

The following keywords were selected: “Iran plants”, “Iranian medicinal plants”, “Iranian plants biological activities”. Only articles in the English language were selected, and data from patents, symposiums, and congress abstracts were excluded because not enough complete to warrant an effective comparison with full papers. Papers that did not show a clear botanical identification were rejected. The database www.theplantlist.org was used to check the correctness of the nomenclature of the reported plant species.

## 3. Results and Discussion

### 3.1. Medicinal Plants Traditionally Used in Iran

Iran has a history of great importance in the field of traditional medicine practices; this knowledge heritage goes back to the time of Babylonian-Assyrian civilization; every generation added his experience and new elements to this “cultural database”. Nowadays, medicinal plants are still used in Iran as curatives for various types of health problems [4]. A great part of this traditional knowledge has not been considered by the scientific point of view yet, and it would be advisable to check the effectiveness of the traditional treatments, especially when there are no supporting data in the scientific literature.

A bibliographic search was performed, selecting ethnobotanical studies conducted through questionnaires and personal interviews with traditional healers and local people in the Iranian territory that include clear botanical identification of the plants, traditional uses, and type of administration.

Table 1 collects reports of plants used for medicinal purposes in the Iranian territory, their local name, the part of the plant used, type of extraction/preparation, the territory where the plant use is reported.

### 3.2. Biological Activities of Plants Grown and Collected in Iran

A bibliographic search was conducted, focusing on biological activities of plants collected in the Iranian territory. The purpose of this section is to collect data related to scientific studies in order to evidence potential correlations between traditional treatments and proved biological activities of plants and phytocomplexes obtained from them. The results are summarized in Table 2.

#### 3.2.1. Antibacterial Activity

Abedini et al. (2014) [13] tested the antimicrobic activity of forty-four methanolic extracts, obtained from plants grown and collected in the Iranian territory, against thirty-five pathogenic bacteria and one yeast. The biological activity was evaluated with Müller–Hinton agar in Petri dishes seeded by a multiple inoculator and minimal inhibition concentration (MIC) method. The authors identified four candidates that deserve further chemical characterization and biological evaluation: *Dorema ammoniacum, Ferula assa-foetida, Ferulago contracta* (Seeds), and *Perovskia abrotanoides* (Aerial parts). These plants showed broad-spectrum activity and interesting MIC values against one or several strains (MIC= 78 μg/mL). The lowest MIC value of 78 μg/mL was achieved by *Dorema ammoniacum* against *Staphylococcus aureus*, *Staphylococcus epidermidis*, *Staphylococcus lugdunensis. Ferula assa-foetida* against *Staphylococcus aureus* and *Staphylococcus epidermidis. Ferulago contracta* against *Staphylococcus epidermidis* [13].

Bonjar (2004) [11] evaluated the antibacterial properties of forty-five plant species used in Iranian traditional medicine practices against eleven bacterial species. The extracts were prepared by maceration of the plant material with methanol for three days, and the result was lyophilized after filtration. The lyophilized methanol extracts were diluted to a concentration of 20 mg/mL in dimethylsulfoxide (DMSO): methanol (1/1: *v*/*v*) solvent in order to perform antimicrobial bioassay. The author declared that the following plant extracts showed broad spectra antimicrobial activity:

*Rhus coriaria* L., *Trachyspermum ammi* L., *Alhagi maurorum* Medik., *Trigonella foenum-graecum* L., *Lawsonia inermis* L., *Rheum ribes* L., and *Cuminum cyminum* L. Further studies are needed to find out which compounds are responsible for this activity. Particular plants, such as *Lawsonia inermis* L., which is active against *Pseudomonas fluorescens* and *Trachyspermum ammi* L., *Nymphaea alba* L. active against *Pseudomonas aeruginosa*, are proper candidates for further studies as possible sources of active compounds [11].

Chitsazian-Yazdi et al. (2015) [15] studied an Iranian medicinal plant known for its various biological activities, including antispasmodic and anthelmintic, named *Ferula foetida* Regel (Apiaceae).

Sulfur compounds obtained by methanolic extract of the roots of the plant were isolated and characterized to test their antimicrobial activity and cytotoxic activity. Six compounds were isolated: foetithiophene C, foetithiophene F, foetithiophene A, foetithiophene B, coniferaldehyde, and sinapic aldehyde.

Their antimicrobial activities and cytotoxicity were evaluated using broth microdilution method and Alamar blue assay. Antimicrobial activity was evidenced against Gram-positive bacteria, more in particular foetithiophene F, which showed interesting antimicrobial activity with MIC value 50 mg/mL against the Gram-positive *Bacillus cereus.* No cytotoxic activity was detected against MCF-7 and K562 cells [15].

Koochak et al. (2010) [12] conducted a preliminary study regarding the antibacterial activity of ethanolic extracts obtained by four plant species used in traditional medicinal practices in Iran. The studied plants were *Beta vulgaris* L., *Amaranthus graecizans* L., *Rumex obtusifolius* L., *Polygonum patulum* M. Bieb. The antibacterial activity was tested using the agar disc diffusion method against Gram-positive and Gram-negative bacteria. No one of the used extracts had significant antibacterial activity against Gram-negative bacteria. The highest activity was evidenced by the ethanolic extract of *Polygonum patulum* against *Streptococcus pyogenes* (inhibitory zone = 28 mm) followed by *Beta vulgaris* against *Staphylococcus epidermidis* (inhibitory zone = 23 mm) and *Rumex obtusifolius* against *Streptococcus pyogenes*. Minimum inhibitory concentration (MIC) = minimum bactericidal concentration (MBC) = 5 mg/mL. Further studies are needed to define which compounds contained in the extracts are responsible for the antimicrobial activity [12].

Lotfipour et al. (2008) [10] tested the antimicrobial activity of thirty-six extracts obtained from ten plans collected in north-west Iran against some Gram-negative strains.

Among them, the methanol extract of *Thalictrum minus* was the most active one with a minimum inhibitory concentration (MIC) value of 0.3125 mg/mL against *Staphylococcus aureus*.

Furthermore, the broad spectra of activity of some plant extracts (especially methanolic extracts) studied, obtained by the plants *Thalictrum minus*, *Salvia sahendica*, *Achillea millefollum*, and *Echium italicum*, were promising [10].

Mehdi Razavi et al. (2011) [16] tested the in vitro antimicrobial activity and cytotoxic activity of different extracts obtained by the plant *Malva sylvestris* L. (flowers and leaves); this plant is commonly used in traditional medicine practices in Iran. Flowers and leaves of the plant were collected from Tabriz, Iran. The flowers methanolic extract showed high antibacterial effects against some human pathogenic bacteria strains, such as *Staphylococcus aureus*, *Streptococcus agalactiae*, *Enterococcus faecalis*, with MIC values of 192, 200, and 256 μg/mL, respectively. Further studies are needed to identify the main active compounds [16].

Nariman et al. (2004) [18] tested the antibacterial activity of six plants collected (and endemic) in Iran, against *Helicobacter pylori*: *Glycyrrhiza aspera*, *Juglans regia*, *Ligustrum vulgare*, *Thymus kotschyanus*, *Trachyspermum copticum*, and *Xanthium brasilicum.* A disk susceptibility assay was used for the evaluation. All of the studied extracts showed anti-*H. pylori* activity; the most active were obtained from *Xanthium brasilicum* and *Trachyspermum copticum;* the solvents used to obtain the extracts were water and an equal mixture of methanol, petroleum benzene, diethyl ether. Minimum inhibitory concentrations (MIC) of the extracts obtained from the two plants range from 31.25 to 250 μg/mL [18].

Pirbalouti et al. (2010) [17] tested the antibacterial activity of essential oils and ethanolic extracts obtained by ten plants traditionally used as medicaments grown and collected in Iran. The tested vegetal extracts were investigated against *Staphylococcus aureus*, *Escherichia coli*, *Pseudomonas aeruginosa*, and *Klebsiella pneumoniae* by agar disc diffusion assay. Most of the samples showing antibacterial activity were considered as interesting by the authors against the tested bacteria with the diameter of inhibition zone ranging between 8 and 23 mm. The most interesting plants were *Satureja bachtiarica* and *Thymus daenensis* (leaves and flowers), with MIC values ranging from 0.039 to 10 mg/mL [17].

Sepahi et al. (2014) [14] tested the antibacterial activity of aqueous extracts obtained by four plants collected in Iran: *Ferula gummosa, Echinophora orientalis, Nasturtium microphyllum*, and *Verbascum thapsus*. The radial diffusion assay was performed using *Staphylococcus aureus* and *Escherichia coli*; moreover, hemolysis assay was used to test eventual toxic effects on human red blood cells. All the studied extracts showed interesting activity with MIC values lower than 750 μg/mL, and these extracts deserve further studies to identify the main active compounds [14].

#### 3.2.2. Antifungal Activity

Imani et al. (2015) [20] studied the essential oil obtained by hydro-distillation of aerial parts of *Zhumeria majdae*, which is a traditionally used medicinal plant endemic in Iran. The antifungal activity was determined using the serial dilution method. The essential oil (EO) was tested on six pathogenic fungal species and one yeast, and all of them resulted as sensitive to *Z. majdae* essential oil. Moreover, in particular, the essential oil was interestingly effective against *Candida albicans*, with a MIC (minimal inhibitory concentration) of 0.031 μL/mL. This evidence confirmed the value of *Zhumeria majdae* as an antifungal agent, and further studies are needed to identify the compounds responsible for this biological activity [20].

In a study conducted by Pirbalouti et al. (2009) [19], the anti-*Candida* activity of essential oils and extracts of nine plants grown and collected in Iran was tested by agar disc diffusion assay. The studied plants are used in ethnomedical practices. Most of the tested samples showed diameters of inhibition zone ranging from 7 to 46 mm; moreover, in particular, the extracts of *Ziziphus spinachristi* and *Scrophularia striata* and the essential oil of *Satureja bachtiarica* showed the best anti-*Candida* activity, followed by the essential oils of *Thymus daenensis* and *Trachyspermum ammi* [19].

#### 3.2.3. Antimalarial Activity

Afshar et al. (2018) [22] studied the in vitro antimalarial activity of different extracts of three Iranian endemic species belonging to the *Scrophularia* genus, including *Scrophularia frigida*, *Scrophularia subaphylla*, and *Scrophularia atropatana*. The antimalarial activity was tested by the cell-free β-hematin formation assay. Among the studied extracts, the dichloromethane one, obtained by aerial parts of *Scrophularia frigida*, exhibited strong antimalarial activity with inhibitory capacity (IC_50_) value of 0.67 ± 0.11 mg/mL. *Scrophularia frigida* represented a deserving candidate for further studies focused on the identification of the main active compounds [22].

Feiz Haddad et al. (2017) [21] tested the in vitro and in vivo antimalarial activity of ten Iranian plants used in traditional medicine practices. All the plants’ samples were collected in the Iranian territory. Methanolic extracts were tested for in vitro antimalarial activity against chloroquine-sensitive 3D7 and multi-drug resistant K1 strains of *Plasmodium falciparum*. The in vivo activity against *Plasmodium berghei* infection in mice was determined. *Citrullus colocynthis* fruits, *Physalis alkekengi* leaves and fruits, and *Solanum nigrum* fruits displayed potent in vitro antimalarial activity against both 3D7 and K1 strains; the in vivo studies comparisons between mice treated with the three plant extracts and untreated controls showed reduced parasitemia by 65.08%, 57.97%, and 60.68%, respectively [21]. Moreover, no toxicity was evidenced. Further studies can be designed to identify the active constituents and clarify their mechanism of action.

#### 3.2.4. Antioxidant Activity

Alinezhad et al. (2012) [25] tested the antioxidant activity of ethyl acetate extracts of stems and leaves and owes of the plant *Hyssopus angustifolius*, collected in Iran. Antioxidant activity of extracts was evaluated with six different tests: nitric oxide, hydrogen peroxide scavenging, 2,2-diphenyl-1-picrylhydrazyl (DPPH), metal chelating, reducing power activities, and hemoglobin-induced linoleic acid system. The results confirmed the interesting antioxidant profile of this plant; it could be a natural source of active compounds. Further studies are necessary to identify the main active compounds present in the different parts of the plant [25].

Dehghan et al. (2016) [23] evaluated the antioxidant activity and α-amylase and α-glucosidase inhibition activity of n-hexane, ethyl acetate, and methanolic extracts obtained by various parts of eleven plants grown and collected in Hyrcania region, Iran. As regards the antioxidant activity, methanolic extract of *Convolvulus persicus* roots (IC_50_ = 38.9 mg/mL), methanolic extract of *Pyrus boissieriana* stems (IC_50_ = 39.3 mg/mL), and methanolic extract of *Primula heterochroma* leaves, and ethyl acetate and methanolic extracts of its roots (IC_50_ = 41.7 mg/mL, 37.9mg/mL, and 30.1 mg/mL, respectively) evidenced strong activity if compared with butylated hydroxytoluene (BHT) (IC_50_ = 16.7 mg/mL; used as a positive control) [23].

Dehghan et al. (2017) [24] evaluated the antioxidant and antidiabetic activity of extracts obtained by the plant *Heracleum persicum.* This work led to the isolation of eleven furanocoumarins. These compounds were identified as psoralen, bergapten, xanthotoxin, iso-pimpinellin, angelicin, isobergapten, sphondin, pimpinellin, heratomin, 5-methoxyheratomin, moellendorffiline, and fraxetin. As the antioxidant activity concerns, among the listed compounds, moellendorffiline exhibited strong antioxidant activity with IC_50_ = 0.2 μM, a value that was interesting if compared with butylated hydroxytoluene (BHT) (IC_50_ = 0.1 µM; used as a positive control) [24]. 

Ebrahimabadi et al. (2010) [30] tested the antioxidant activity of polar and non-polar fractions of the methanolic extract obtained by the plant *Stachys inflata*. Aerial parts of the plant were collected from Kashan area, Isfahan province, Iran. The biological activity was tested using 2,2-diphenyl-1-picrylhydrazyl (DPPH) and β-carotene/linoleic acid assays. In the DPPH test, interesting results were shown by the methanolic extract polar subfraction with an IC_50_ of 89.50 μg/ mL, indicating an antioxidant potency of about 22% of that of butylated hydroxytoluene (IC_50_ = 19.72 μg/mL). In β-carotene/linoleic acid assay, the best inhibition belonged to the nonpolar subfraction, with an inhibition percentage of 77.08%. Further studies are needed to identify the main active compounds [30].

Fathiazad et al. (2011) [26] studied the ethyl acetate and n-butanol extracts obtained by aerial parts of the plant *Hyssopus officinalis* L., a medicinal herb collected from north of Iran. Total phenolic content and antioxidant activity were tested by Folin–Ciocalteau and DPPH tests. Apigenin 7-O-β-D-glucuronide was also isolated as the major flavon. Phenolic content of n-butanol and ethyl acetate extracts was determined and expressed as milligrams of gallic acid equivalents—246 mgGAE/g and 51 mg GAE/g, respectively. The antioxidant activity of apigenin 7-O-β-D-glucuronide, ethyl acetate extract, and the n-butanol extract was determined, obtaining IC_50_ values of 116×10^−3^, 103×10^−3^, 25×10^−3^ mg/mL, respectively. The purified apigenin 7-O-β- D-glucuronide showed weak activity. The extract that showed interesting antioxidant activity values, because of the highest content of total phenolic compounds, was the n-butanol one [26].

Khazaeli et al. (2009) [28] tested five traditional medicinal plants from Iran on free radicals scavenging activity and on the inhibition of mushroom tyrosinase activity. Focusing on the radical scavenging activity, methanolic extracts of *Quercus infectoria* and *Terminalia chebula* showed a strong radical scavenging effect in the 2,2′-diphenyl-1-picrylhydrazyl (DPPH) assay with values of IC_50_ (concentration providing 50% inhibition of the DPPH radical) of 15.3 and 82.2 μg/mL, respectively. This study encouraged further investigations on *Quercus infectoria* and *Terminalia chebula* in the field of solar protection (due to the radical scavenging activity) and of skin depigmentation agents (due to inhibitory effects on mushroom tyrosinase) [28].

Dehshiri et al. (2013) [31] tested the antioxidant activity of laminas, stems, petioles, fruits, peduncles, and flowers in the hydro-alcoholic extracts from the plant *Tetrataenium lasiopetalum.* The plant samples were collected from Oshtoran Kuh, Azna, Lorestan, Iran. Antioxidant activities of the extracts were examined by different in vitro assays: 2,2′-diphenyl-1-picrylhydrazyl (DPPH) radical scavenging, metal chelating, reducing power activities, and hemoglobin-induced linoleic acid system. All the tested extracts showed interesting antioxidant activity, confirming hypotheses based on traditional knowledge. Moreover, in particular, the hydro-alcoholic extract of the flower showed the highest activity in the DPPH test (IC_50_ = 170 ± 7 μg/mL). In the metal chelating assay, lamina extract showed the best iron ion chelating activity among the other extracts (IC_50_ = 230 ± 10 μg/mL). Lamina hydro-alcoholic extract demonstrated better activity in the hemoglobin-induced linoleic acid system test than other parts of *T. lasiopetalum* [31]. Further studies could identify the main active compounds.

Pourmorad et al. (2006) [27] worked on the antioxidant activity, phenol, and flavonoid content of five plants (*Mellilotus officinalis*, *Equisetum maximum*, *Plantago major*, *Adiantum capillus-veneris*, and *Urtica dioica*) collected from Northern provinces of Iran (Gilan and Mazandaran). Methanolic extraction was performed after drying at room temperature, and the result was freeze-dried. The extract of *Mellilotus officinalis* showed a high amount of flavonoid (57 ± 5.4 mg/g) and phenolic compounds (289.5 ± 5 mg/g) and exhibited the greatest radical scavenging activity (IC_50_ = 0.018 mg/mL) in a DPPH test among the tested extracts [27].

Sonboli et al. (2010) [29] assessed antioxidant activities and total phenolic contents of methanolic extracts obtained from male inflorescences of *Salix aegyptiaca* L., grown and collected in Ashena Abad village, Urmia (West Azarbaijan province), Iran. 2,2-diphenyl-1-picrylhydrazyl (DPPH) free radical scavenging assay and Folin–Ciocalteu method were performed on the whole methanolic extract and on three fractions (water fraction, butanol fraction, and chloroform fraction) obtained from it. The butanol fraction evidenced, among the others, the best antioxidant activity and the highest phenolic content with an IC_50_ value of 27.7 μg/ mL and total phenols of 313.8 ppm; the results were interesting because this extract was comparable with the synthetic antioxidant butylated hydroxytoluene (BHT) (IC_50_ = 26.5 μg/mL) [29]. The detected antioxidant activity encouraged the use of this plant for its antioxidant properties in food industries and in cosmetic and pharmaceutical preparations.

#### 3.2.5. Anticancer Activity/Cytotoxic Activity

Asadi-Samani et al. (2018) [33] tested the in vitro antiproliferative activity of twenty Iranian medicinal plants against prostate cancer. The plant samples were collected in Chaharmahal and Bakhtiari provinces, Iran. The extraction of the powdered aerial parts was conducted by maceration in ethanol 70% for 72 h and was concentrated under reduced pressure. Antiproliferative activity of the tested extracts on PC-3, DU145 (prostate cancer cell lines), and HDF (non-cancer cell line) cell lines was evaluated by MTT (3-[4,5-dimethylthiazol-2-yl]-2,5 diphenyl tetrazolium bromide) assay. The hydro-alcoholic extract obtained by the plant *Euphorbia szovitsii* Fisch. & C. A. Mey showed good antiproliferative activity against PC-3 and DU145 cell lines. *Urtica dioica* and *Medicago sativa* resulted active only on the DU-145 cell line. These results could be a starting point in the development of new anticancer drugs, and further studies are needed in order to identify the main active compounds [33].

Esmaeilbeig et al. (2015) [36] tested the in vitro anticancer activities of ten species of plants grown and collected in southern Iran using the MTT colorimetric assay. Methanolic extracts obtained by aerial parts of the plants *Arctium lappa, Cichorium intybus*, *Glycyrrhiza glabra, Alhagi psuedalhahi*, *Mentha longifolia*, *Thymus daenensis*, *Thymus vulgaris*, *Satureja bachtiarica*, *Satureja hortensis*, and *Rheum ribes* were tested against five tumor cell lines: K562 (myelogenous leukemia), Jurkat (T cell leukemia), and Raji (Burkitt’s lymphoma), Fen (bladder carcinoma), and HeLa (human cervical epithelioid carcinoma). No activity was detected against solid tumor cell lines Fen and HeLa, and leukemic cell lines demonstrated to be more sensitive to the extracts. *Satureja hortensis*, *Satureja bachtiarica*, *Thymus vulgaris*, *Thymus daenensis*, and *Mentha longifolia* showed strong inhibitory activity on Jurkat cells with inhibition values higher than 80% at a concentration of 200 μg/mL. At the same concentration, these extracts inhibited the K562 cell line with more than 50% of inhibition [36]. Further studies are needed to identify the main active compounds.

Hamzeloo-Moghadam et al. (2015) [35] tested the cytotoxic activity and the apoptosis induction activity of different fractions obtained by methanolic extract of *Hypericum scabrum* leaves. The plant was collected from Alborz province, Iran. The petroleum ether, dichloromethane, and methanol fractions were evaluated for cytotoxicity against M-CF7, A-549, HT-29, and HepG-2 cell lines. The apoptosis induction ability was assessed by activated caspase-3 inspection and Annexin V FITC/PI (propidium iodide) assays.

The results evidenced strong cytotoxicity against HT-29 and HepG-2 cell lines and interesting apoptosis induction ability; the authors suggested further studies in this field [35].

Jassbi et al. (2016) [32] tested the cytotoxic activity, against three human cancer cell lines (LS180, MCF-7, and MOLT-4), of dichloromethane and methanol extracts of *Anthemis mirheydari*, an endemic plant from Iran. The plant samples were collected in Jahrom in Fars province, Iran, and the whole plant was used for the extraction. The dichloromethane extract evidenced interesting IC_50_ values, 30.8, 25.2, and 8.6 mg/mL for the three cell lines, respectively. Four compounds were isolated from the dichloromethane extract: taraxasterol, pseudotaraxasterol, β-sitosterol, and 7-methoxycoumarin. Taraxasterol and 7-methoxycoumarin are known in scientific literature to present anticancer properties; this fact, along with the encouraging results of the study, makes *Anthemis mirheydari* a new potential anticancer medicinal plant that certainly deserves further investigations [32].

Mehdi Razavi et al. 2011 [16] tested the in vitro cytotoxic activity of different extracts obtained by the plant *Malva sylvestris* L. (flowers and leaves); this plant is commonly used in traditional medicine practices in Iran. Flowers and leaves of the plant were collected from Tabriz, Iran. The methanolic extracts of flowers and leaves evidenced interesting cytotoxic activity against the MacCoy cell line, reducing their viability with IC_50_ values of 265.3 and 311.0 μg/mL, respectively. The authors declared that *Malva sylvestris* L. plant extracts could be considered as an antiproliferative agent [16]. Further studies are needed to identify the main active compounds.

Sahranavard et al. (2009) [34] tested the cytotoxic activity of methanolic extracts of fifteen Iranian medicinal plants against three cancer cell lines (MCF7, HepG2, WEHI164). The extract obtained by *Ferula szowitsiana* root showed IC_50_ values lower than 100 μg/mL in all the tested cell lines, and it was chosen for further studies. Fractionation was performed, which led to the isolation of two monoterpenoids; both of them were bornyl esters that were identified as Chimganin and Chimgin. These compounds showed interesting cytotoxic effects with values of IC_50_ significantly lower if compared to the whole extract; they performed a little less than tamoxifen, which was used as a positive control. These results demonstrated that the two compounds were mostly responsible for the cytotoxic activity of this plant [34].

#### 3.2.6. Antidiabetic Activity

Dehghan et al. (2016) [23] evaluated α-amylase and α-glucosidase inhibition activities of n-hexane, ethyl acetate, and methanol extracts obtained by various parts of eleven plants grown and collected in Hyrcania region, Iran.

The n-hexane extract of *Heracleum persicum* (aerial parts, roots), ethyl acetate and n-hexane extract of *Smilax excelsa* (stem and leaves), methanolic, n-hexane, ethyl acetate extract of *Pyrus boissieriana* (leaves and steam), ethyl acetate and methanolic extract of *Parrotia persica* (leaves), and methanolic and ethyl acetate extract of *Primula heterochroma* (leaves and roots) exhibited significant antidiabetic activities in α- glucosidase and α-amylase assays, more effective than acarbose used as a positive control [23]. These plants, in conclusion, are deserving candidates for further studies in the antidiabetic field.

Dehghan et al. (2017) [24] evaluated the antidiabetic activity of extracts obtained by the plant *Heracleum persicum.* This work led to the isolation of eleven furanocoumarins. These compounds were identified as psoralen, bergapten, xanthotoxin, iso-pimpinellin, angelicin, isobergapten, sphondin, pimpinellin, heratomin, 5-methoxyheratomin, moellendorffiline, and fraxetin. Among them, moellendorffiline showed significant inhibitory activity against α-glucosidase with an IC_50_ value of 17.9 nM, and it was more active than acarbose (IC_50_ = 23.5 nM; used as a positive control) [24].

Hasanein et al. (2016) [37] studied the effects of *Salvia officinalis* L. against learning and memory deficit induced by diabetes. This plant has been used in Iranian traditional medicine practices against diabetes. The plant samples were collected in Hamedan, Iran. The effects of the leaves’ hydro-alcoholic extract on passive avoidance learning (PAL) and memory in streptozocin-induced diabetic and non-diabetic rats were evaluated. Administration for thirty days demonstrated to alleviate the negative influence of diabetes on learning and memory. Positive effects on hyperglycemia and oxidative stress were evidenced. Therefore, *Salvia officinalis* L. and its constituent rosmarinic acid represented a potential therapeutic option against diabetic memory impairment, and further studies are needed to clarify the mechanisms involved in this activity [37].

#### 3.2.7. Iron chelating Activity

Ebrahimzadeh et al. (2008) [38] tested the iron chelating activity, phenol, and flavonoid content of eleven medicinal plants from Iran. The extraction was performed by maceration of the vegetal dried material for three days. The solvent was evaporated under reduced pressure and then lyophilized. *Epilobium hirsutum* leaves and *Melilotus arvensis* showed the best chelating activity with IC_50_ values of 0.49 ± 0.01 mg/mL and 0.08 ± 0.01 mg/mL, respectively. These plant extracts also showed high phenol and flavonoid contents. *Feijoa sellowiana* leaves and *Pistacia lentiscus* showed good chelating activity [38].

#### 3.2.8. Anti-Platelet Aggregation Activity

Lorigooini et al. (2014) [39] studied the essential oil obtained by aerial parts of *Allium atroviolaceum.* The plant was collected in Rig mountain, Shahr-e-kord province, Iran. In this work, the anti-platelet aggregation activity of the essential oil was examined using arachidonic acid (AA) and adenosine diphosphate (ADP) as platelet aggregation inducers.

The essential oil evidenced dose-dependent inhibitory effect against AA and ADP-induced aggregation with IC_50_ values of 0.25 mg/mL and 0.47 mg/mL, respectively [39]. Further studies are required to identify the main active compounds of the essential oil.

#### 3.2.9. Mushroom Tyrosinase Inhibition Activity

Khazaeli et al. (2009) [28] tested five traditional medicinal plants from Iran on the inhibition of mushroom tyrosinase activity. Methanolic extracts obtained from *Quercus infectoria* galls and *Terminalia chebula* fruits showed inhibitory effects on mushroom tyrosinase in the hydroxylation of L-tyrosine (85.9% and 82.2% of inhibition, respectively). Furthermore, these two plants inhibited the oxidation of Levodopa (L-DOPA), performing similarly to kojic acid (used as a positive control) with values of IC_50_ = 102.8 and 192.6 μg/mL, respectively [28]. This study encouraged further investigations on the two plants in the field of solar protection due to the radical scavenging activity and of skin depigmentation agents due to inhibitory effects on mushroom tyrosinase.

#### 3.2.10. Acetylcholinesterase-Inhibitory Activity

Abbas-Mohammadi et al. (2018) [42] tested the acetylcholinesterase-inhibitory activity of n-hexane, ethyl acetate, and methanolic extracts obtained by aerial parts of twenty-five plants grown and collected in Iran. The evaluation was conducted by an in vitro enzymatic Ellman method and molecular docking study. The n-hexane extract obtained by the plant *Prangos ferulacea* showed the highest acetylcholinesterase (AChE)-inhibitory activity with 75.6% inhibition at a concentration of 50 μg/mL. The chemical characterization of the extract led to the identification of seventeen compounds. Further studies led to the identification of a subfraction (named F_10f_) that resulted as the most potent inhibitor of AChE in this extract with an IC_50_ value of 25.2 μg/mL [42]. *Prangos ferulacea* deserves further in vivo and in vitro studies as the discovery of new acetylcholinesterase (AChE) inhibitors might lead to new tools for the treatment of Alzheimer’s disease.

Adhami et al. (2011) [41] tested the acetylcholinesterase-inhibitory activity of forty herbal drugs traditionally used against cognitive disorders in Iran. Eighty drugs were tested by TLC bioautography method and microplate colorimetric assay, and, due to the interesting activity, the seeds of *Peganum harmala* L. were investigated in detail. The alkaloids harmaline and harmine were identified as active compounds. The IC_50_ values were 8.4 μg/mL for harmaline (pure compound) and 10.9 μg/mL for harmine (pure compound), 41.2 μg/mL for the methanolic extract, 95.5 μg/mL for the dichloromethane extract [41]. The two tested alkaloids were the major AChE-inhibitory compounds in *Peganum harmala*; this plant deserves further studies to test the biological activity in vivo.

Jazayeri et al. (2014) [40] evaluated the acetylcholinesterase-inhibitory activity of eighteen aqueous-methanolic extracts (1:1 v/v) obtained by plants commonly used in Iranian traditional medicine collected in Tehran. The inhibitory activity was tested using the in vitro Ellman spectrophotometric method. According to the results, five plants evidenced interesting properties. The inhibitory activity values, expressed as IC_50_ μg/mL, in fact were 5.96 μg/mL for *Camellia sinensis* (leaves), 19.57 μg/mL for *Citrus aurantifolia* (fruits), 24.37 μg/mL for *Zizyphus vulgaris* (fruits), 84.30 μg/mL for *Brassica nigra* (seeds), and 93.1 μg/mL for *Rosa damascena* (flowers) [40]. Further investigations regarding the identification of active components in the extracts are needed.

#### 3.2.11. Antihyperlipidemic and Antihypertensive Activities

Asgary et al. (2000) [43] studied the antihyperlipidemic and antihypertensive effects of *Achillea wilhelmsii* C. Koch drops, with a double-blind placebo-controlled clinical trial. The aerial parts of the plant were collected in Chatrood village in the province of Kerman, Southeast Iran. Moderate hyperlipidemic and primary hypertensive subjects were treated with a hydro-alcoholic extract twice daily for more than six months. The results showed a significant decrease in triglycerides after two months of treatment. Significant decreases in triglycerides, total cholesterol, and low-density lipoproteins (LDL)-cholesterol were observed after four months of treatment. Levels of high-density lipoproteins (HDL)-cholesterol were significantly increased after six months. A significant decrease in diastolic and systolic blood pressure was observed after two and six months, respectively [43].

#### 3.2.12. Gastric Antiulcerogenic Activity

Karimi et al. (2004) [44] studied the gastric antiulcerogenic activity of aqueous and ethanolic extracts obtained from the plant *Portulaca oleracea* L. collected in the village of Khaje-rabi, Khorasan province, Iran. Both leaves extracts, tested in vivo in mice, showed remarkable dose-dependent inhibition of gastric lesions induced by absolute ethanol or HCl [44]. This gastroprotective activity resulted in line with Iranian traditional medicine knowledge, and it deserves further studies to determine the involved mechanisms. 

#### 3.2.13. Anti-Dyspepsia Activity

Khonche et al. (2017) [45] tested the efficacy of *Mentha pulegium* L., collected in the Alborz province of Iran, against functional dyspepsia in a randomized double-blind placebo-controlled clinical trial. Leaves of this plant are used in Iranian traditional medicine practices to treat dyspeptic symptoms. The hydro-alcoholic leaf extract taken daily for two months was shown to be effective in the reduction of dyspeptic symptoms, improving quality of life, and contributing to eradicate *Helicobacter pylori* in patients affected by functional dyspepsia [45].

#### 3.2.14. Inhibitory Effect on Gastric Acid Output

Niazmand et al. (2010) [46] studied the effects of the aqueous-ethanolic extract obtained by aerial parts of the plant *Achillea wilhelmsii* on rat’s gastric acid output in basal, vagotomized, and vagal-stimulated conditions. The plant samples were collected from South Khorasan province, Iran. *Achillea wilhelmsii* is a plant frequently used in Iranian traditional medicine against gastrointestinal disorders. The results of the in vivo study showed that the aqueous-ethanol extract of *A. wilhelmsii* exhibited an inhibitory effect on gastric acid output in basal conditions via the gastric parasympathetic nerve. The extract had no effect on vagal-stimulated conditions [46]. Further studies are needed to identify the compounds and mechanisms responsible for this activity.

#### 3.2.15. Anti-Colitic Activity

Minaiyan et al. (2011) [47] tested the anti-colitic activity of hydro-alcoholic extract and the essential oil obtained by *Rosmarinus officinalis* leaves. The plant material was collected in the city of Isfahan, Iran. The study was performed in vivo on a model of experimental colitis induced by trinitrobenzene sulfonic acid in rats.

Both the extracts at all the tested doses demonstrated to be effective in the reduction of colon tissue lesions and of colitis indices; the higher doses tested were considerably effective in diminishing histopathologic parameters. These data supported the traditional medicine knowledge and suggested that both hydro-alcoholic extract and the essential oil obtained by *Rosmarinus officinalis* leaves possess consistent anti-colitic activity [47].

## 4. Conclusions

The Iranian territory possesses a great abundance of plants suitable for medicinal use and remarkable heritage of knowledge handed down from generation to generation concerning natural remedies against a wide range of diseases and disorders. Nowadays, the study of this heritage is at an early stage.

As reported in Section 3 and Section 4, the bibliographic research evidenced ethnobotanical studies conducted in the Iranian territory, carrying out questionnaires and interviews with traditional healers or local people, and scientific studies inspired by traditional medicinal practices conducted on plants collected in Iran. Comparing ethnobotanical studies and traditional medicine-inspired scientific studies, it is evident that most of the Iranian traditional herbal remedies have not been considered from a scientific point of view yet. Only 34 plants are cited in both Section 3 and Section 4 among the 245 of Section 3. Table 3 provides a comparison between traditional uses and tested biological activities of the plants cited both in Section 3 and Section 4.

The identification of a direct correspondence between the traditional uses and biological activities represents a complex issue. Some plant species mentioned in this work have already been studied in other parts of the world with different climatic characteristics and, consequently, different phytocomplexes. In our opinion, it is of interest to study plants that are not interesting from a medicinal point of view in other parts of the world if included in traditional medicinal practices in Iran, as they could be active due to a quite different phytocomplex expressed in the particular climatic characteristics and ecosystems of the Iranian territory. It should be pointed out that, considering the research works found in literature, the process of valorization and study of plant species does not often pay particular attention to the aspect of sustainability of eventual systematic exploitation. This aspect is becoming more and more important these days.

Traditional remedies are often effective due to the synergistic activity of a large number of compounds that are part of the plant phytocomplex; therefore, careful research is needed to identify the active molecules. The research work is further complicated by the fact that in some cases, natural remedies act as palliatives. In any case, the evidence that nature has always inspired medicine, constituting itself as a source of inspiration for the development of pharmacological treatments, makes the study of traditional remedies a very important component of basic research in the medicinal and pharmacological field.

A summary of the information in the scientific literature, related to documented traditional medicinal practices and plants studied from a scientific point of view in the same territory, represents a useful tool to plan new researches in order to avoid repeating work already done and to concentrate on apparently effective but not yet scientifically evaluated plants. In our opinion, there is still a large room for scientific works that could deepen the above-stated aspects, encouraging further research in the field.

## Figures and Tables

**Table 1:** Plants traditionally used as medicinal remedies in the Iranian territory. Local name, part of the plant used, type of extraction/preparation, the area where the use of the plant is reported. (N.r. = not reported).

	Scientific Name	Family	Local Name	Part Used	Type of Extract	Medicinal Uses	Area	Author (s)
***1.***	*Abelmoschus esculentus* (L.) Moench	Malvaceae	Bamieh	Seed	n.r.	Anti-inflammatory, Diuretic, Laxative	Mashhad city, Northeastern Iran	[5] Amiri and Joharchi 2013
***2.***	*Acanthophyllum sordidum* Bunge ex Boiss.	Caryophylaceae	Choobak	Root	n.r.	Warts, Washing	Mashhad city, Northeastern Iran	[5] Amiri and Joharchi 2013
***3.***	*Acanthophyllum spp.*	Caryophyllaceae	Chobak	Aerial parts	Herbal tea/decoction	Antiparasitic	Shiraz, Fars province	[6] Bahmani et al. 2016
***4.***	*Achillea millefolium* L. *Achillea millefolium L.*	Asteraceae Asteraceae	n.r. Boomadaran	Inflorescence Aerial parts	Boiled, steamed Herbal tea/decoction	Antidiabetic Antiparasitic	Urmia county, Northwest Iran Shiraz, Fars province	[7] Bahmani et al. 2014 [6] Bahmani et al. 2016
***5.***	*Achillea santolinoides subsp. wilhelmsii* (K. Koch) Gruter	Asteraceae	Bumadaran	Aerial parts	n.r.	Anti-hemorrhoids, Antidiarrhea, Hypoglycemic, Anthelmintic, Mastitis, Antacid, Dyspepsia, Nerve Tonic, Treatment of Osteoarthritis, Treatment of Blood Flooding, Appetizer	Mashhad city, Northeastern Iran	[5] Amiri and Joharchi 2013
***6.***	*Adiantum capillus-veneris* L.	Pteridaceae	Parsiavashan	Aerial parts	n.r.	Antitussive, Anti-hemorrhoid, Treatment of Sore Throat, Febrifuge, Jaundice, Laxative, Anti-thirst, Treatment of Orchitis	Mashhad city, Northeastern Iran	[5] Amiri and Joharchi 2013
***7.***	*Alcea spp.*	Malvaceae	Gole Khatmi	Flower	n.r.	Antitussive, Febrifuge, Treatment of Pimples, Laxative, Depurative, Treatment of Gum Swelling	Mashhad city, Northeastern Iran	[5] Amiri and Joharchi 2013
***8.***	*Alhagi graecorum* Boiss.	Fabaceae	Taranjabin	Manna	n.r.	Jaundice, Laxative, Febrifuge, Thirst, Aphthous Ulcers	Mashhad city, Northeastern Iran	[5] Amiri and Joharchi 2013
***9.***	*Alhagi maurorum* Medik.	Fabaceae	Khar Shotor- Taranjabin	Aerial parts - Manna	n.r.	Appetite Suppressant, Diuretic, Jaundice, Febrifuge	Mashhad city, Northeastern Iran	[5] Amiri and Joharchi 2013
***10.***	*Allium altissimum* Regel	Amaryllidaceae	Musir	Bulb	n.r.	Antiseptic, Appetizer, Digestive	Mashhad city, Northeastern Iran	[5] Amiri and Joharchi 2013
***11.***	*Allium cepa* *Allium cepa* L.	Amaryllidaceae Amaryllidaceae	Piaz Piaz	Bulb Seed	Herbal tea/decoction n.r.	Antiparasitic Treatment of Trichoptlosis	Shiraz, Fars province Mashhad city, Northeastern Iran	[6] Bahmani et al. 2016 [5] Amiri and Joharchi 2013
***12.***	*Allium haementhoides* Bioss. & Ruet. Ex Regel	Liliaceae	Sorpa	Leaf, flower stem	Brew	Peptic Ulcer	Lorestan province	[8] Delfan et al. 2015
***13.***	*Allium sativum* L.	Amaryllidaceae	Sir	Bulb	n.r.	Hypoglycemic, Cardiac Diseases, Antiseptic, Toothache, Antihyperlipidemia, Anthelmintic, Antihypertensive	Mashhad city, Northeastern Iran	[5] Amiri and Joharchi 2013
***14.***	*Althaea officinalis* L.	Malvaceae	Charme giah	Root	n.r.	Mouth Wounds, Bone Fracture, Treatment of Bruises, Treatment of Dysuria	Mashhad city, Northeastern Iran	[5] Amiri and Joharchi 2013
***15.***	*Alyssum alyssoides* (L.) L.	Brassicaceae	Ghodumeh	Seed	n.r.	Pharyngitis, Antitussive, Febrifuge, Laxative, Treatment of Hoarseness	Mashhad city, Northeastern Iran	[5] Amiri and Joharchi 2013
***16.***	*Alyssum desertorum* Stapf.	Brassicaceae	n.r.	Seed	Boiled, herbal fumigation	Antidiabetic	Urmia county, Northwest Iran	[7] Bahmani et al. 2014
***17.***	*Amaranthus caudatus* L.	Amaranthaceae	Taj Khorus	Aerial parts	n.r.	Disinfectant Treatment of Enteritis, Febrifuge, Antitussive, Antidiarrhea, Laxative	Mashhad city, Northeastern Iran	[5] Amiri and Joharchi 2013
***18.***	*Amygdalus commonis* *Amygdalus communis*	Rosaceae Rosaseae	Badam-e shirin Baadam	Green fruit and seed Fruit	Boiled, brewed, raw Herbal tea/decoction	Anti-hair Loss Antiparasitic	Khiregah-e Jangali, Ghasemloo Shiraz, Fars province	[9] Baharvand-Ahmadi et al. 2015 [6] Bahmani et al. 2016
***19.***	*Anacamptis morio* (L.) R. M. Bateman	Orchidaceae	Saalab gholveh	Root	n.r.	Tonic	Mashhad city, Northeastern Iran	[5] Amiri and Joharchi 2013
***20.***	*Anastatica hierochuntica* L.	Brassicaceae	Change mayam	Aerial parts	n.r.	Bring Luck to Pregnant Women, Menstrual Regulator	Mashhad city, Northeastern Iran	[5] Amiri and Joharchi 2013
***21.***	*Anchusa italica*	Boraginaceae	Gole-gazou	Leaf, flower	Decoction	Stomach Ache	Lorestan province	[8] Delfan et al. 2015
***22.***	*Anethum graveolens* L	Apiaceae	Shevid	Fruit	n.r.	Abortion, Anti-dysmenorrhea, Galactogogue, Antihyperlipidemia, Carminative	Mashhad city, Northeastern Iran	[5] Amiri and Joharchi 2013
***23.***	*Anthemis tinctoria* L.	Asteraceae	Baboone-ye zard	Flowering shoot	Boiled, brewed, paste	Beauty and Clarity of the Skin, Strengthening of Hair Roots	Khiregah-e Jangali, Ghasemloo valley	[9] Baharvand-Ahmadi et al. 2015
***24.***	*Apium graveolens* L.	Apiaceae	Karafs	Fruit	n.r.	Emmenagogue, Diuretic, Carminative	Mashhad city, Northeastern Iran	[5] Amiri and Joharchi 2013
***25.***	*Arctium lappa* L. *Arctium lappa* L.	Asteraceae Asteraceae	Baba Adam n.r.	Leaves - Root Root, leaf	n.r. Boiled, steamed	Diuretic Cholagogue, Depurative, Hypoglycemic Antidiabetic	Mashhad city, Northeastern Iran Urmia county, Northwest Iran	[5] Amiri and Joharchi 2013 [7] Bahmani et al. 2014
***26.***	*Arnebia euchroma* (Royle) I.M.Johnst.	Boraginaceae	Havachoobeh	Root	n.r.	Treatment of Dermal Disorders, Hair Tonic	Mashhad city, Northeastern Iran	[5] Amiri and Joharchi 2013
***27.***	*Artemisia absinthium* *Artemisia absinthium* L.	Asteraceae Asteraceae	Ofsantin Afsantin	Leaf Aerial parts	Herbal tea/decoction n.r.	Antiparasitic Anthelmintic, Appetizer, Indigestion	Shiraz, Fars province Mashhad city, Northeastern Iran	[6] Bahmani et al. 2016 [5] Amiri and Joharchi 2013
***28.***	*Artemisia dracunculus* L.	Asteraceae	Tarkhun	Leaves	n.r.	Appetizer, Dyspepsia, Anthelmintic, Antacid, Carminative	Mashhad city, Northeastern Iran	[5] Amiri and Joharchi 2013
***29.***	*Artemisia sieberi* Besser	Asteraceae	Dermaneh	Flowering shoot	Boiled, brewed, paste	Baldness	Khiregah-e Jangali, Ghasemloo valley	[9] Baharvand-Ahmadi et al. 2015
***30.***	*Artemisia vulgaris* L.	Asteraceae	Baranjasef	Flower	n.r.	Nerve Tonic, Sexual Impotency, Menstrual Regulator	Mashhad city, Northeastern Iran	[5] Amiri and Joharchi 2013
***31.***	*Arundo donax* L.	Poaceae	Tabashir ghalam	Latex	n.r.	Aphthous Ulcer, Anti Thirst, Depurative, Treatment of Pimples, Febrifuge	Mashhad city, Northeastern Iran	[5] Amiri and Joharchi 2013
***32.***	*Astragalus adscendens* Boiss. & Hausskn. ex Boiss.	Fabaceae	Gazangabin	Manna	n.r.	Laxative, Febrifuge Digestive	Mashhad city, Northeastern Iran	[5] Amiri and Joharchi 2013
***33.***	*Astragalus fasciculifolius* subsp. *arbusculinus* (Bornm. & Gauba) Tietz	Fabaceae	Anzerut	Gum	n.r.	Antitussive, Jaundice, Laxative, Anthelmintic	Mashhad city, Northeastern Iran	[5] Amiri and Joharchi 2013
***34.***	*Astragalus hamosus* L.	Fabaceae	Nakhonak	Fruit	n.r.	Anodyne, Repel of Kidney Stone, Diuretic, Arthrodynia, Carminative	Mashhad city, Northeastern Iran	[5] Amiri and Joharchi 2013
***35.***	*Astragalus* sieversianus Pall.	Fabaceae	Gol Sefid	Fruit	n.r.	Menstrual Disorders	Mashhad city, Northeastern Iran	[5] Amiri and Joharchi 2013
***36.***	*Astragalus spp.*	Fabaceae	Katira	Gum	n.r.	Mouth Wounds, Aphrodisiac, Cystitis, Hair Tonic	Mashhad city, Northeastern Iran	[5] Amiri and Joharchi 2013
***37.***	*Atropa belladonna* L.	Solanaceae	Beladon	Leaves	n.r.	Antispasmodic, Sedative	Mashhad city, Northeastern Iran	[5] Amiri and Joharchi 2013
***38.***	*Avena sativa* L. *Avena sativa* L.	Poaceae Poaceae	Jo dosar n.r.	Seed Seed, glumelle	n.r. Boiled	Treatment of Acne Blood Refining	Mashhad city, Northeastern Iran Urmia county, Northwest Iran	[5] Amiri and Joharchi 2013 [7] Bahmani et al. 2014
***39.***	*Berberis integerima* Bunge. *Berberis integerima* Bunge	Berberidaceae Berberidaceae	n.r. Zereshk Kuhi	Fruit, leaf, skin Fruit	Boiled, steamed n.r.	Antidiabetic Hypoglycemic, Antihypertensive, Blood and Liver Cleanser, Jaundice, Febrifuge, Antigout	Urmia county, Northwest Iran Mashhad city, Northeastern Iran	[7] Bahmani et al. 2014 [5] Amiri and Joharchi 2013
***40.***	*Berberis sp.*	Berberidaceae	Zereshk	Fruit	n.r.	Antigout, Blood and Liver Cleanser, Febrifuge, Anthelmintic, Treatment of Dysentery	Mashhad city, Northeastern Iran	[5] Amiri and Joharchi 2013
***41.***	*Borago officinalis*	Boraginaceae	Gholegavzaban	Flower	Herbal tea/decoction	Antiparasitic	Shiraz, Fars province	[6] Bahmani et al. 2016
***42.***	*Brassica napus* *Brassica napus* L.	Brassicaceae Brassicaceae	Kolza Shalgham	Leaf Seed	Decoction n.r.	Stomach Ache Antiseptic, Treatment of Cold, Tonic	Lorestan province Mashhad city, Northeastern Iran	[8] Delfan et al. 2015 [5] Amiri and Joharchi 2013
***43.***	*Brassica nigra* (L.) K.Koch	Brassicaceae	Khardal	Seed	n.r.	Laxative	Mashhad city, Northeastern Iran	[5] Amiri and Joharchi 2013
***44.***	*Bunium cylindricum* (Boiss. & Hohen.) Drude	Apiaceae	Zireh Siah	Fruit	n.r.	Carminative	Mashhad city, Northeastern Iran	[5] Amiri and Joharchi 2013
***45.***	*Bunium persicum* (Boiss.) B. Fedtsch.	Apiaceae	Zireh Siah	Fruit	n.r.	Obesity, Galactogogue, Flavoring, Carminative, Calmative, Appetizer, Indigestion	Mashhad city, Northeastern Iran	[5] Amiri and Joharchi 2013
***46.***	*Caccinia macranthera* (Banks & Sol.) Brand	Boraginaceae	Gavzaban sabz	Aerial parts	n.r.	Sedative, Treatment of Cough, Expectorant	Mashhad city, Northeastern Iran	[5] Amiri and Joharchi 2013
***47.***	*Camellia sinensis* (L.) Kuntze	Theaceae	Chai Sabz	Leaves	n.r.	Obesity, Anticancer, Antihypertensive, Hepatitis, Antihyperlipidemia	Mashhad city, Northeastern Iran	[5] Amiri and Joharchi 2013
***48.***	*Cannabis sativa* L.	Cannabinaceae	Shahdaneh	Seed	n.r.	Sedative, Tonic Treatment of Osteoarthritis, Treatment of Ear Pain	Mashhad city, Northeastern Iran	[5] Amiri and Joharchi 2013
***49.***	*Capparis spinosa* L.	Capparaceae	Kavar	Fruit-Root	n.r.	Liver Tonic, Hepatitis, Appetizer, Anthelmintic, Stomach Tonic, Emmenagogue, Antigout	Mashhad city, Northeastern Iran	[5] Amiri and Joharchi 2013
***50.***	*Capsella bursa-pastoris* (L.) Medik.	Brassicaceae	Kiseh Keshish	Seed	n.r.	Period Regulator, Anti-hemorrhage, Antidiarrhea	Mashhad city, Northeastern Iran	[5] Amiri and Joharchi 2013
***51.***	*Capsicum annuum* L.	Solanaceae	Felfel Ghermez	Fruit	n.r.	Appetizer, Spice, Treatment of Osteoarthritis, Tonic, Stimulant, Aphrodisiac	Mashhad city, Northeastern Iran	[5] Amiri and Joharchi 2013
***52.***	*Carthamus tinctorius* L.	Asteraceae	Golrang (Kajireh)	Flower - Seed	n.r.	Emmenagogue, Flavoring Luxative, Treatment of Rheumatism	Mashhad city, Northeastern Iran	[5] Amiri and Joharchi 2013
***53.***	*Centaurea behen* L.	Asteraceae	Bahman Sefid	Root	n.r.	Aphrodisiac, Anti-lithiasis	Mashhad city, Northeastern Iran	[5] Amiri and Joharchi 2013
***54.***	*Centaurea depressa* M. Bieb.	Asteraceae	Gole Gandom	Aerial parts	n.r.	Digestive, Febrifuge, Cholagogue, Blood Cleanser, Antigout	Mashhad city, Northeastern Iran	[5] Amiri and Joharchi 2013
***55.***	*Cerasus avium* (L.) Moench	Rosaceae	Dome Gilas	Pedicel	n.r.	Anti-lithiasis, Prostate Disorders Kidney Stone, Anti-inflammatory	Mashhad city, Northeastern Iran	[5] Amiri and Joharchi 2013
***56.***	*Cerasus microcarpa*	Rosaceae	n.r.	Fruit	Boiled, raw use	Blood Refining	Urmia county, Northwest Iran	[7] Bahmani et al. 2014
***57.***	*Ceterach officinalis*	Phillicineae	Sarakhs	Aerial parts	Paste	Head Itching	Khiregah-e Jangali, Ghasemloo valley	[9] Baharvand-Ahmadi et al. 2015
***58.***	*Cichorium intybus* L. *Cichorium intybus* L.	Asteraceae Asteraceae	Kasni Kasni	Aerial parts Root, leaves, flower, and seeds	n.r. Boiled	Treatment of Palpitation, Appetizer, Depurative, Treatment of Furuncles, Jaundice, Febrifuge, Anti-allergic Head Itching	Mashhad city, Northeastern Iran Khiregah-e Jangali, Ghasemloo valley	[5] Amiri and Joharchi 2013 [9] Baharvand-Ahmadi et al. 2015
***59.***	*Cinnamomum verum* *Cinnamomum verum*	Lauraceae Lauraceae	n.r. Darchin	Skin Fruit shells	Boiled Herbal tea/decoction	Antidiabetic Antiparasitic	Urmia county, Northwest Iran Shiraz, Fars province	[7] Bahmani et al. *2014* [6] Bahmani et al. 2016
***60.***	*Citrulluscolocynthis* (L.) Schrad. *Citrullus colocynthis* (L.) Schrad.	Cucurbitaceae Cucurbitaceae	n.r. Hanzal	Fruit Fruit-Seed	Boiled n.r.	Antidiabetic Purgative, Anodyne, Hypoglycemic	Urmia county, Northwest Iran Mashhad city, Northeastern Iran	[7] Bahmani et al. *2014* [5] Amiri and Joharchi 2013
***61.***	*Citrus aurantiifolia* (Christm.) Swingle	Rutaceae	Limu Amani	Fruit	n.r.	Antihypertensive, Calmative	Mashhad city, Northeastern Iran	[5] Amiri and Joharchi 2013
***62.***	*Citrus aurantium* L.	Rutaceae	Bahar Naranj	Flower	n.r.	Anti-stress, Cardiac Tonic, Food Digestion, Antihypertensive	Mashhad city, Northeastern Iran	[5] Amiri and Joharchi 2013
***63.***	*Clinopodium graveolens* (M. Bieb.) Kuntze	Lamiaceae	Faranjmeshk	Seed	n.r.	Pharyngitis, Gastric Ulcer, Nerve Tonic	Mashhad city, Northeastern Iran	[5] Amiri and Joharchi 2013
***64.***	*Colchicum autumnale* L.	Colchicaceae	Suranjan	Root	n.r.	Antigout, Calmative, Arthrodynia	Mashhad city, Northeastern Iran	[5] Amiri and Joharchi 2013
***65.***	*Colchicum kotschyi* Boiss.	Liliaceae	Gol-e hasrat	Flower	Paste	Lice	Khiregah-e Jangali, Ghasemloo valley	[9] Baharvand-Ahmadi et al. 2015
***66.***	*Conium maculatum* L.	Apiaceae	Shokaran	Root	n.r.	Cholagogue, Depilator, Treatment of Dermal Allergies	Mashhad city, Northeastern Iran	[5] Amiri and Joharchi 2013
***67.***	*Convolvulus arvensis* L.	Convolvulaceae	Pichak-e sahraee	Aerial parts	Paste	Skin Spots	Khiregah-e Jangali, Ghasemloo valley	[9] Baharvand-Ahmadi et al. 2015
***68.***	*Cordia myxa* L.	Boraginaceae	Sepestan	Fruit	n.r.	Pharyngitis, Antitussive, Febrifuge, Laxative	Mashhad city, Northeastern Iran	[5] Amiri and Joharchi 2013
***69.***	*Coriandrum sativum* L.	Apiaceae	Geshniz	Fruit	n.r.	Acne, Treatment of Flatulence, Appetizer, Aphrodisiac, Calmative, Jaundice, Antiseptic, Aromatic	Mashhad city, Northeastern Iran	[5] Amiri and Joharchi 2013
***70***	*Cornus mas* L.	Cornaceae	Zoghal Akhteh	Fruit	n.r.	Prostatic Hypertrophy, Anti-hemorrhage, Antidiarrhea, Febrifuge	Mashhad city, Northeastern Iran	[5] Amiri and Joharchi 2013
***71.***	*Coronilla varia* L.	Fabaceae	n.r.	Leaf	Raw use, boiled	Antidiabetic	Urmia county, Northwest Iran	[7] Bahmani et al. 2014
***72.***	*Corylus avellana* L.	Betulaceae	Fandogh	Fruit	n.r.	Treatment of Anemia, Depurative, Appetizer	Mashhad city, Northeastern Iran	[5] Amiri and Joharchi 2013
***73.***	*Crataegus aronia* (L.) Bosc ex Dc.	Rosaceae	n.r.	Fruit and skin	Raw use, boiled	Antidiabetic	Urmia county, Northwest Iran	[7] Bahmani et al. 2014
***74.***	*Crataegus oxycantha* L.	Rosaceae	n.r.	Fruit, flower root, skin	Raw use, boiled	Antidiabetic	Urmia county, Northwest Iran	[7] Bahmani et al. 2014
***75.***	*Crataegus sp.*	Rosaceae	Sorkhe Valik	Fruit-Leaves	n.r.	Depurative, Repairs Blood Vessel	Mashhad city, Northeastern Iran	[5] Amiri and Joharchi 2013
***76.***	*Crocus sativus* L.	Iridaceae	Zaffaron	Style	n.r.	Tonic, Dysmenorrheal, Emmenagogue, Nerve Tonic, Premature Ejaculation, Gastric Ulcer, Aphrodisiac	Mashhad city, Northeastern Iran	[5] Amiri and Joharchi 2013
***77.***	*Cucumis sativus* L.	Cucurbitaceae	Khiar	Seed	n.r.	Diuretic, Anti-lithiasis, Blood Cleansing, Febrifuge	Mashhad city, Northeastern Iran	[5] Amiri and Joharchi 2013
***78.***	*Cuminum cyminum* L.	Apiaceae	Zireh Sabz (Keravieh)	Fruit	n.r.	Treatment of Colic, Galactogogue, Obesity, Digestive, Flavoring, Antiseptic	Mashhad city, Northeastern Iran	[5] Amiri and Joharchi 2013
***79.***	*Cuscuta epithymum* Murray	Convolvolaceae	Aftimun	Aerial parts	n.r.	Laxative, Anti-hemorrhoids	Mashhad city, Northeastern Iran	[5] Amiri and Joharchi 2013
***80.***	*Cydonia oblonga* Mill.	Rosaceae	Beh Daneh	Seed-Leaves	n.r.	Cardiac Diseases, Antitussive, Sore Throat, Laxative, Febrifuge	Mashhad city, Northeastern Iran	[5] Amiri and Joharchi 2013
***81.***	*Cyperus rotundus* L.	Cyperaceae	Soade Kufi	Root	n.r.	Strengthening of Memory	Mashhad city, Northeastern Iran	[5] Amiri and Joharchi 2013
***82.***	*Dactylorhiza umbrosa* (Kar. & Kir.) Nevski	Orchidaceae	Saalab panjeh	Root	n.r.	Treatment of Sexual Impotency, Tonic	Mashhad city, Northeastern Iran	[5] Amiri and Joharchi 2013
***83.***	*Datura stramonium* L. *Datura stramonium* L.	Solanaceae Solanaceae	Tatureh Tatureh	Seed Seed	n.r. Boiled and Paste	Sedative, Treatment of Addiction, Treatment of Colic Wound Healing, Wound Disinfection	Mashhad city, Northeastern Iran Khiregah-e Jangali, Ghasemloo valley	[5] Amiri and Joharchi 2013 [9] Baharvand-Ahmadi et al. 2015
***84.***	*Daucus carota* L.	Apiaceae	Havij	Fruit	n.r.	Diuretic, Emmenagogue	Mashhad city, Northeastern Iran	[5] Amiri and Joharchi 2013
***85.***	*Delphinium semibarbatum* Bien. ex Boiss	Ranunculaceae	Zarir	Flower	n.r.	Treatment of Dermal Allergies, Coloring	Mashhad city, Northeastern Iran	[5] Amiri and Joharchi 2013
***86.***	*Descurainia Sophia (L.) Schr.* *Descurainia sophia* (L.) Webb ex Prantl	Brassicaceae Brassicaceae	Khakeshir Khakshir	Fruit Seed	Fresh food n.r.	Antiparasitic Blood and Liver Cleanser, Jaundice, Febrifuge, Treatment of Furuncles, Anti-thirst, Laxative	Shiraz, Fars province Mashhad city, Northeastern Iran	[6] Bahmani et al. 2016 [5] Amiri and Joharchi 2013
***87.***	*Dorema ammoniacum* D. Don	Apiaceae	Kandal	Gum- Root	n.r.	Cystitis, Digestive, Treatment of Colic, Treatment of Furuncles, Expectorant, Anthelmintic, Emmenagogue, Anticovulsion	Mashhad city, Northeastern Iran	[5] Amiri and Joharchi 2013
***88.***	*Drimia maritima* (L.) Stearn	Asparagaceae	Onsol	Bulb	n.r.	Arthrodynia, Emmenagogue, Hair Tonic	Mashhad city, Northeastern Iran	[5] Amiri and Joharchi 2013
***89.***	*Dysphania botrys* (L.) Mosyakin & Clemants	Amaranthaceae	Dermaneh Torki	Aerial parts	n.r.	Diabetes, Treatment of Sinusitis, Respiratory Disorders, Anthelmintic, Antacid, Antidiarrhea, Carminative, Urinary Antiseptic	Mashhad city, Northeastern Iran	[5] Amiri and Joharchi 2013
***90.***	*Echinops cephalotes* DC.	Asteraceae	Shekar Tighal	Manna	n.r.	Antitussive, Anti-asthmatic, Pharyngitis, Febrifuge	Mashhad city, Northeastern Iran	[5] Amiri and Joharchi 2013
***91.***	*Echium amoenum* Fisch. & C.A.Mey.	Boraginaceae	Gole Gavzaban	Flower	n.r.	Antihypertensive, Nerve Tonic, Diuretic, Anti-stress, Blood Cleanser Cardiac Tonic	Mashhad city, Northeastern Iran	[5] Amiri and Joharchi 2013
***92.***	*Elaeagnus angustifolia* L.	Elaeagnaceae	Senjed	Fruit	n.r.	Arthrodynia, Antidiarrhea, Treatment of Rheumatism, Female Aphrodisiac	Mashhad city, Northeastern Iran	[5] Amiri and Joharchi 2013
***93.***	*Ephedra major* Host	Ephedraceae	Khakestar Koshtar	Aerial parts	n.r.	Treatment of Joints Pain	Mashhad city, Northeastern Iran	[5] Amiri and Joharchi 2013
***94.***	*Equisetum arvense* L. *Equisetum arvense* L. *Equisetum arvense* L.	Equisetaceae Equisetaceae Equisetaceae	Dome Asb Dome Asb n.r.	Aerial parts Aerial parts Aeration organ	n.r. Boiled Boiled	Obesity, Anti-lithiasis, Antihypertensive, Prostate Disorders, Treatment of kidney Disorders Hair Loss, Nails Strengthening Antidiabetic	Mashhad city, Northeastern Iran Khiregah-e Jangali, Ghasemloo valley Urmia county, Northwest Iran	[5] Amiri and Joharchi 2013 [9] Baharvand-Ahmadi et al. 2015 [7] Bahmani et al. 2014
***95.***	*Eremurus spectabilis* M. Bieb.	Xanthorrhoeaceae	Serish	Root	n.r.	Dermal Infection, Sticking, Antihyperlipidemia	Mashhad city, Northeastern Iran	[5] Amiri and Joharchi 2013
***96.***	*Eruca sativa* (L.) Mill.	Brassicaceae	Mandab (Roghan cheragh)	Seed	n.r.	Sedative, Laxative Diuretic, Stomach Tonic,	Mashhad city, Northeastern Iran	[5] Amiri and Joharchi 2013
***97.***	*Euphorbia macroclada* Boiss.	Euphorbiaceae	Ferfion	Leaves	Paste	Wart	Khiregah-e Jangali, Ghasemloo valley	[9] Baharvand-Ahmadi et al. 2015
***98.***	*Falcaria vulgaris* Bernh.	Apiaceae	Ghaz Yaghi	Leaves - Fruit	n.r.	Treatment of Vitiligo, Cut, Wound	Mashhad city, Northeastern Iran	[5] Amiri and Joharchi 2013
***99.***	*Ferula assa foetida L* *Ferula foetida* (Bunge) Regel	Apiaceae Apiaceae	Anghozeh Anghuzeh	Leaf Gum	Herbal tea/decoction n.r.	Antiparasitic Anthelmintic, Treatment of Colic, Emmenagogue	Shiraz, Fars province Mashhad city, Northeastern Iran	[6] Bahmani et al. 2016 [5] Amiri and Joharchi 2013
***100.***	*Ferula gummosa* Boiss.	Apiaceae	Barijeh	Gum- Root	n.r.	Anthelmintic Anticatarrhal, Anti-allergic, Dyspepsia, Appetizer, Emmenagogue	Mashhad city, Northeastern Iran	[5] Amiri and Joharchi 2013
***101.***	*Ficus carica* L.	Moraceae	Anjir	Fruit	n.r.	Anti-hemorrhoids, Laxative, Tonic	Mashhad city, Northeastern Iran	[5] Amiri and Joharchi 2013
***102.***	*Ficus johannis Boiss.*	Moraceae	Anjirevahshi	Fruit	Fresh food and herbal tea/decoction	Antiparasitic	Shiraz, Fars province	[6] Bahmani et al. 2016
***103.***	*Foeniculum vulgare* Mill. *Foeniculum vulgaris*	Apiaceae Apiaceae	Razianeh Raziane	Fruit Seed	n.r. Decoction	Galactogogue, Digestive, Bronchitis, Appetizer, Antacid, Flatulence Peptic Ulcer	Mashhad city, Northeastern Iran Lorestan province	[5] Amiri and Joharchi 2013 [8] Delfan et al. 2015
***104.***	*Fraxinus excelsior* L.	Oleaceae	Zaban Gonjeshk	Fruit	n.r.	Aphrodisiac, Treatment of Stammering	Mashhad city, Northeastern Iran	[5] Amiri and Joharchi 2013
***105.***	*Fritillaria imperialis* L.	Liliaceae	Laleh Sarnegun	Root	n.r.	Treatment of Joints Pain	Mashhad city, Northeastern Iran	[5] Amiri and Joharchi 2013
***106.***	*Fumaria asepala* Boiss.	Fumariaceae	Shahtareh	Aerial parts	Boiled	Head and Face Itching, Allergy, Face Acne	Khiregah-e Jangali, Ghasemloo valley	[9] Baharvand-Ahmadi et al. 2015
***107.***	*Fumaria vaillantii* Loisel.	Papaveraceae	Shatareh	Aerial parts	n.r.	Pimples, Febrifuge, Blood Cleansing, Psoriasis, Appetizer, Antiacid, Jaundice	Mashhad city, Northeastern Iran	[5] Amiri and Joharchi 2013
***108.***	*Gentiana olivieri* Griseb.	Gentianaceae	Suloo	Flower	n.r.	Cardiac Ailments	Mashhad city, Northeastern Iran	[5] Amiri and Joharchi 2013
***109.***	*Glycyrrhiza glabra* L.	Fabaceae	Shirin Bayan	Root	n.r.	Antitussive, Antacid, Tonic, Gastric Ulcer, Treatment of Hypotension, Treatment of Anemia	Mashhad city, Northeastern Iran	[5] Amiri and Joharchi 2013
***110.***	*Gundelia tournefortii* L.	Asteraceae	Kangar	Aerial parts	n.r.	Liver Tonic, Treatment of Hepatitis	Mashhad city, Northeastern Iran	[5] Amiri and Joharchi 2013
***111.***	*Gundeliatournefortii*	Asteraceae	Kanghar	Fruit	Fresh food	Antiparasitic	Shiraz, Fars province	[6] Bahmani et al. 2016
***112.***	*Helichrysum graveolens* (M. Bieb.) Sweet	Asteraceae	Afsantin	Aerial parts	n.r.	Anodyne, Anthelmintic, Appetizer, Nerve Tonic	Mashhad city, Northeastern Iran	[5] Amiri and Joharchi 2013
***113.***	*Heracleum persicum* *Heracleum persicum* Desf.	Apiaceae Apiaceae	Kolpar Golpar	Leaf, flower Fruit	Decoction n.r.	Stomach Ache Treatment of Hiccup, Appetizer, Flavoring, Carminative, Anthelmintic, Stomach Tonic	Lorestan province Mashhad city, Northeastern Iran	[8] Delfan et al. 2015 [5] Amiri and Joharchi 2013
***114.***	*Hibiscus syriacus* L.	Malvaceae	Gole Khatmi	Flower	n.r.	Febrifuge, Antitussive	Mashhad city, Northeastern Iran	[5] Amiri and Joharchi 2013
***115.***	*Hibiscus trionum* L.	Malvaceae	Khatmi-e seh rang	Flower	Boiled, boiled and brewed for washing	Head Itching, Strengthening of Hair Root	Khiregah-e Jangali, Ghasemloo valley	[9] Baharvand-Ahmadi et al. 2015
***116.***	*Humulus lupulus* L.	Cannabinaceae	Razak	Hops	n.r.	Diuretic, Treatment of Sleeplessness, Kidney Tonic, Calming, Sedative for Digestion	Mashhad city, Northeastern Iran	[5] Amiri and Joharchi 2013
***117.***	*Hymenocrater spp.*	Lamiaceae	Badranjbuyeh	Aerial parts	n.r.	Cardiac Tonic, Hypnotic, Antitussive, Carminative, Dyspnoea, Anti-stress Convulsion	Mashhad city, Northeastern Iran	[5] Amiri and Joharchi 2013
***118***	*Hyoscyamus niger* L.	Solanaceae	Bangdaneh	Seed	n.r.	Sedative, Treatment of Addiction, Treatment of Toothache, Treatment of Headache, Antigout	Mashhad city, Northeastern Iran	[5] Amiri and Joharchi 2013
***119.***	*Hypecum pendulum*	Apiaceae	Shah tare	flowering shoot	Boiled	Skin Allergy	Khiregah-e Jangali, Ghasemloo valley	[9] Baharvand-Ahmadi et al. 2015
***120.***	*Hypericum scabrum* L.	Hypericaceae	Hufarighun	Flower	n.r.	Antimigraine, Gastric Ulcer, Anti hemorrhage, Urinary Incontinence, Treatment of Headache	Mashhad city, Northeastern Iran	[5] Amiri and Joharchi 2013
***121.***	*Indigofera argentea* Burm.f.	Fabaceae	Rang	Leaves	n.r.	Antifungal, Hair Color, Hair Tonic	Mashhad city, Northeastern Iran	[5] Amiri and Joharchi 2013
***122.***	*Iris spuria* L.	Iridaceae	Zanbagh	Root	n.r.	Arthrodynia, Diuretic	Mashhad city, Northeastern Iran	[5] Amiri and Joharchi 2013
***123.***	*Ixillirion tataricum* (Pall.) Roem et Schult	Amaryllidaceae	Khiarak	Gland, flowering shoot	Paste	Washing of Skin Abscess and Disinfection of Infectious Wounds	Khiregah-e Jangali, Ghasemloo valley	[9] Baharvand-Ahmadi et al. 2015
***124.***	*Juglans regia* *Juglans regia* *Juglans regia* L.	Juglandaceae Juglandaceae Juglandaceae	Gerdou n.r. Gerdu	Fruit, trunk palm, leaves Fruit, leaf, and skin Fruit- Leaves	Boiled Boiled n.r.	Anti-allergic, Hematopoietic Antidiabetic Eczema, Antidiarrhea, Hair Color	Khiregah-e Jangali, Ghasemloo valley Urmia county, Northwest Iran Mashhad city, Northeastern Iran	[9] Baharvand-Ahmadi et al. 2015 [7] Bahmani et al. 2014 [5] Amiri and Joharchi 2013
***125.***	*Juniperus sabina* L.	Cupressaceae	Abhal	Fruit	n.r.	Diuretic, Anti-lithiasis, Food Digestion, Urinary Antiseptic	Mashhad city, Northeastern Iran	[5] Amiri and Joharchi 2013
***126.***	*Lamium album* L.	Lamiaceae	n.r.	Flowering offshoot	Boiled	Antidiabetic	Urmia county, Northwest Iran	[7] Bahmani et al. 2014
***127.***	*Lactuca sativa* L.	Asteraceae	Kahu	Seed	n.r.	Anti-thirst, Hypnotic	Mashhad city, Northeastern Iran	[5] Amiri and Joharchi 2013
***128.***	*Lagenaria vulgaris*	Cucurbitaceae	Kadoo	Seed	Herbal tea/decoction	Antiparasitic	Shiraz, Fars province	[6] Bahmani et al. 2016
***129.***	*Lallemantia iberica* (M.Bieb.) Fisch. & C.A. Mey.	Lamiaceae	Tokhm Sharbati	Seed	n.r.	Gastric Ulcer, Antitussive, Laxative, Hoarseness, Anti-thirst	Mashhad city, Northeastern Iran	[5] Amiri and Joharchi 2013
***130.***	*Laurus nobilis* L.	Lauraceae	Barg Bu	Leaves	n.r.	Carminative Appetizer, Flavor	Mashhad city, Northeastern Iran	[5] Amiri and Joharchi 2013
***131.***	*Lawsonia inermis* L.	Lythraceae	Hana	Leaves	n.r.	Hair Color, Treatment of Headache, Hair Tonic, Washing, Antifungal, Antiseptic	Mashhad city, Northeastern Iran	[5] Amiri and Joharchi 2013
***132.***	*Lepidium sativum* L.	Brassicaceae	Shahi (Tartizak)	Seed	n.r.	Appetizer, Anthelmintic, Laxative, Sore Throat	Mashhad city, Northeastern Iran	[5] Amiri and Joharchi 2013
***133.***	*Levisticum officinale* W.D.J.Koch	Apiaceae	Angedane roomi	Fruit	n.r.	Nerve Diseases, Heart Tonic, Indigestion	Mashhad city, Northeastern Iran	[5] Amiri and Joharchi 2013
***134.***	*Linum usitatissimum* L. *Linum usititassimum* L.	Linaceae Linaceae	Katan Katan	Seed Seed	n.r. Boiled	Cholesterol-lowering, Antitussive, Laxative, Obesity Bedsore	Mashhad city, Northeastern Iran Khiregah-e Jangali, Ghasemloo valley	[5] Amiri and Joharchi 2013 [9] Baharvand-Ahmadi et al. 2015
***135.***	*Malva neglecta* Wallr.	Malvaceae	Nan Kalagh	Flower - Fruit	n.r.	Sore Throat, Antitussive, Febrifuge	Mashhad city, Northeastern Iran	[5] Amiri and Joharchi 2013
***136.***	*Malva sylvestris* L.	Malvaceae	Panirak (Khatmi khabbzi)	Flower - Fruit	n.r.	Pharyngitis, Furuncles, Aphthous Ulcers, Febrifuge, Antitussive, Jaundice, Laxative, Gastric Ulcer, Treatment of Wounds	Mashhad city, Northeastern Iran	[5] Amiri and Joharchi 2013
***137.***	*Marrubium vulgare* L.	Lamiaceae	Ferasion	Aerial parts	n.r.	Liver Tonic, Antitussive	Mashhad city, Northeastern Iran	[5] Amiri and Joharchi 2013
***138.***	*Matricaria chamomilla* L.	Asteraceae	Gole babooneh	Flower	n.r.	Eczema, Antitussive, Anticatarrhal, Hair Tonic, Treatment of Colic, Menstrual Pains	Mashhad city, Northeastern Iran	[5] Amiri and Joharchi 2013
***139.***	*Matricaria recutita*	Asteraceae	Babooneh	Flower, leaf	Herbal tea/decoction	Antiparasitic	Shiraz, Fars province	[6] Bahmani et al. 2016
***140.***	*Medicago sativa* L.	Fabaceae	Yunjeh	Aerial parts	n.r.	Appetizer, Tonic, Osteomalacia, Anti-hemorrhage	Mashhad city, Northeastern Iran	[5] Amiri and Joharchi 2013
***141.***	*Melissa officinalis* L.	Lamiaceae	Badranjbuyeh	Aerial parts	n.r.	Nerve Tonic, Cardiac Tonic, Hypnotic, Antitussive, Carminative, Anti-stress, Convulsion	Mashhad city, Northeastern Iran	[5] Amiri and Joharchi 2013
***142.***	*Mentha longifolia* (L.) Hudson	Lamiaceae	Puneh	Aerial parts	n.r.	Herpes, Anthelmintic, Antacid, Carminative, Antidiarrhea, Digestive	Mashhad city, Northeastern Iran	[5] Amiri and Joharchi 2013
***143.***	*Mentha spicata* L.	Lamiaceae	Naana	Aerial parts	n.r.	Appetizer, Antacid, Carminative, Antidiarrhea, Digestive, Anodyne, Anthelmintic	Mashhad city, Northeastern Iran	[5] Amiri and Joharchi 2013
***144.***	*Morus nigra* L.	Moraceae	Shatut	Root	n.r.	Abortion	Mashhad city, Northeastern Iran	[5] Amiri and Joharchi 2013
***145.***	*Myrtus communis* L.	Myrtaceae	Murd	Leaves - Fruit	n.r.	Psoriasis, Treatment of Sinusitis, Mouth Ulcers, Antifungal, Treatment of Cold, Strengthening of Hair, Herpes	Mashhad city, Northeastern Iran	[5] Amiri and Joharchi 2013
***146.***	*Nasturtium officinale* R. Br.	Brassicaceae	Alafe cheshmeh	Aerial parts	n.r.	Diabetes, Dyspepsia	Mashhad city, Northeastern Iran	[5] Amiri and Joharchi 2013
***147.***	*Nasturtium officinalis* (L.) R. Br.	Cruciferae	n.r.	Leaf, root	Boiled	Antidiabetic	Urmia county, Northwest Iran	[7] Bahmani et al. *2014*
***148.***	*Nepeta binaloudensis* Jamzad	Lamiaceae	Ostokhodus	Aerial parts	n.r.	Treatment of Cold, Carminative, Nerve Tonic, Treatment of Sinusitis, Pulmonary Infections, Treatment of Rheumatism, Anti-asthmatic, Antitussive, Cardiac Tonic	Mashhad city, Northeastern Iran	[5] Amiri and Joharchi 2013
***149.***	*Nepeta bracteata* Benth. *Nepeta bracteata* Benth.	Lamiaceae Lamiaceae	Zufa n.r.	Aerial parts Flowering offshoot	n.r. Boiled, steamed	Pulmonary Infections, Anti-asthmatic, Treatment of cold, Febrifuge, Treatment of Colic, Antitussive Antidiabetic	Mashhad city, Northeastern Iran Urmia county, Northwest Iran	[5] Amiri and Joharchi 2013 [7] Bahmani et al. 2014
***150.***	*Nepeta menthoides* Boiss. & Buhse	Lamiaceae	Ostokhodus	Aerial parts	n.r.	Treatment of Cold, Nerve Tonic, Expectorant	Mashhad city, Northeastern Iran	[5] Amiri and Joharchi 2013
***151.***	*Nepeta meyeri* Benth.	Lamiaceae	n.r.	Flowering offshoot	Boiled, steamed	Antidiabetic	Urmia county, Northwest Iran	[7] Bahmani et al. 2014
***152.***	*Nigella sativa* *Nigella sativa* L.	Ranunculaceae Ranunculaceae	Siah doom Siah Daneh	Seed Seed	Herbal tea/decoction n.r.	Antiparasitic Kidney Stone, Carminative, Antacid, Galactogogue, Anthelmintic, Food Digestion, Antitussive, Treatment of Colic	Shiraz, Fars province Mashhad city, Northeastern Iran	[6] Bahmani et al. 2016 [5] Amiri and Joharchi 2013
***153.***	*Nymphaea alba* L.	Nymphaeaceae	Nilufar Abi	Flower	n.r.	Expectorant, Hypnotic, Antitussive, Calmative	Mashhad city, Northeastern Iran	[5] Amiri and Joharchi 2013
***154.***	*Ocimum basilicum* L.	Lamiaceae	Reyhan (Tokhm sharbati)	Seed	n.r.	Aphthous Ulcers, Antiseptic, Antidiarrhea, Antitussive, Carminative, Laxative, Digestive, Antacid	Mashhad city, Northeastern Iran	[5] Amiri and Joharchi 2013
***155.***	*Origanum vulgare* L.	Lamiaceae	Marzanjush	Aerial parts	n.r.	Treatment of Colic, Treatment of Sinusitis, Sedative, Cardiac Tonic, Nerve Tonic, Treatment of Dyspnoea	Mashhad city, Northeastern Iran	[5] Amiri and Joharchi 2013
***156.***	*Oryza sativa* L.	Poaceae	Chaltooke Berenj	Seed coat	n.r.	Hair Tonic, Treatment of Anemia	Mashhad city, Northeastern Iran	[5] Amiri and Joharchi 2013
***157.***	*Papaver rhoeas* L. *Papaver rhoeas* L.	Papaveraceae Papaveraceae	Shaghayegh n.r.	Flower Seed, capsule	n.r. Boiled	Treatment of Addiction, Calmative, Sleeplessness, Sedative, Expectorant, Antitussive, Anti-asthmatic Antidiabetic	Mashhad city, Northeastern Iran Urmia county, Northwest Iran	[5] Amiri and Joharchi 2013 [7] Bahmani et al. 2014
***158.***	*Papaver somniferum* L.	Papaveraceae	Khashkhash	Fruit- Seed	n.r.	Anodyne, Laxative, Tonic, Hypnotic	Mashhad city, Northeastern Iran	[5] Amiri and Joharchi 2013
***159.***	*Peganum harmala* L.	Nitrariaceae	Espand	Seed	n.r.	Diabetes, Antiseptic, Hypnotic, Treatment of Rheumatism and Sciatica Disorders, Anthelmintic, Emmenagogue	Mashhad city, Northeastern Iran	[5] Amiri and Joharchi 2013
***160.***	*Perovskia abrotanoides* Kar.	Lamiaceae	Gol Kabud	Aerial parts	n.r.	Treatment of Sinusitis, Treatment of Toothache, Antitussive, Nerve Tonic, Carminative, Sedative, Antiseptic, Anthelmintic, Treatment of Colic	Mashhad city, Northeastern Iran	[5] Amiri and Joharchi 2013
***161.***	*Petroselinum crispum* (Mill.) Nyman ex A. W. Hill	Apiaceae	Jafari	Fruit	n.r.	Emmenagogue, Diuretic, Carminative, Kidney Disorders	Mashhad city, Northeastern Iran	[5] Amiri and Joharchi 2013
***162.***	*Phleum pratense L.*	Poaceae	Kalake-gorbe	Branch	Brew	Stomach Ache	Lorestan province	[8] Delfan et al. 2015
***163.***	*Physalis alkekengi* L.	Solanaceae	Arusak Posht Pardeh	Fruit	n.r.	Emmenagogue, Treatment of Kidney Stones, Blood Cleansing	Mashhad city, Northeastern Iran	[5] Amiri and Joharchi 2013
***164.***	*Pimpinella anisum* L.	Apiaceae	Anison (Badian roomi)	Fruit	n.r.	Treatment of Flatulence, Anthelmintic, Treatment of Colic, Antacid, Stomach Ache, Antidiarrhea	Mashhad city, Northeastern Iran	[5] Amiri and Joharchi 2013
***165.***	*Pistacia atlantica* Desf. ssp. kurdica	Anacardiaceae	Saghez	Oleore sin	n.r.	Appetizer, Digestive, Antacid	Mashhad city, Northeastern Iran	[5] Amiri and Joharchi 2013
***166.***	*Pistacia atlantica* Desf. ssp mutica	Anacardiaceae	Baneh	Fruit	n.r.	Laxative, Tonic Stimulant, Treatment of Anemia	Mashhad city, Northeastern Iran	[5] Amiri and Joharchi 2013
***167.***	*Plantago major* L.	Plantaginaceae	Barhang	Seed- Leaves	n.r.	Eczema, Anti-allergic, Febrifuge, Jaundice, Antitussive, Antidiarrhea, Toothache, Depurative, Gastric Ulcer	Mashhad city, Northeastern Iran	[5] Amiri and Joharchi 2013
***168.***	*Plantago ovata* Forssk.	Plantaginaceae	Esfarzeh	Seed	n.r.	Obesity, Depilator, Tonsillitis, Antacid, Antitussive, Gastric Ulcer, Febrifuge, Laxative, Jaundice, Anti-hemorrhoids	Mashhad city, Northeastern Iran	[5] Amiri and Joharchi 2013
***169.***	*Platanus orientalis* L.	Platanaceae	Chenar	Fruit	n.r.	Prostate Diseases	Mashhad city, Northeastern Iran	[5] Amiri and Joharchi 2013
***170.***	*Polygonatum orientale* Desf.	Asparagaceae	Shaghaghol	Root	n.r.	Tonic, Diuretic, Nerve Tonic, Aphrodisiac	Mashhad city, Northeastern Iran	[5] Amiri and Joharchi 2013
***171.***	*Polygonum aviculare* L. *Polygonum aviculare* L.	Polygonaceae Polygonaceae	Alaf Haftband n.r.	Aerial parts Aeration organ	n.r. Boiled	Diabetes, Treatment of Colic, Antidiarrhea Antidiabetic	Mashhad city, Northeastern Iran Urmia county, Northwest Iran	[5] Amiri and Joharchi 2013 [7] Bahmani et al. 2014
***172.***	*Polypodium vulgare* L.	Polypodiaceae	Baspayak	Root	n.r.	Expectorant, Jaundice, Digestive	Mashhad city, Northeastern Iran	[5] Amiri and Joharchi 2013
***173.***	*Portulaca oleracea* L.	Portulacaceae	Khorfeh	Seed- Leaves	n.r.	Antitussive, Febrifuge, Anti-thirst, Food Digestion, Depurative, Diuretic, Anti-hemorrhoids	Mashhad city, Northeastern Iran	[5] Amiri and Joharchi 2013
***174.***	*Punica granatum* *Punica granatum* L.	Punicaceae Punicaceae	Anar-doun Gole Anar	Seed Flower-Root	Pomegranate fruits cooked under hot wood ashes and eat n.r.	Peptic Ulcer Anti-hemorrhage, Blood Flux, Anthelmintic	Lorestan province Mashhad city, Northeastern Iran	[8] Delfan et al. 2015 [5] Amiri and Joharchi 2013
***175.***	*Quercus branti*	Fagaceae	Bali	Pith leaf peel	Oak fruit crushed and mixed with yogurt and eat	Stomach Ache	Lorestan province	[8] Delfan et al. 2015
***176.***	*Quercus infectoria* Oliv.	Fagaceae	Mazuye sabz	Insect gull	n.r.	Nosebleed, Anti-hemorrhage, Uterus Ailments, Mouth Wounds, Anti-hemorrhoids	Mashhad city, Northeastern Iran	[5] Amiri and Joharchi 2013
***177.***	*Quercus spp.*	Fagaceae	Balut (Mazu)	Fruit	n.r.	Antidiarrhea, Anti-hemorrhage	Mashhad city, Northeastern Iran	[5] Amiri and Joharchi 2013
***178.***	*Rheum ribes* L.	Polygonaceae	Rivas	Fruit- Petiole	n.r.	Jaundice, Urinary Antiseptic, Diuretic, Depurative, Liver Tonic, Antiseptic, Hair Tonic	Mashhad city, Northeastern Iran	[5] Amiri and Joharchi 2013
***179.***	*Rheum turkestanicum* Janisch.	Polygonaceae	Eshghan	Root	n.r.	Diabetes, Antihypertensive, Anticancer, Depurative	Mashhad city, Northeastern Iran	[5] Amiri and Joharchi 2013
***180.***	*Rhus coriaria* L. *Rhus coriaria* L.	Anacardiaceae Anacardiaceae	Somagh n.r.	Fruit Fruit, leaf, resin	n.r. Boiled	Jaundice, Cholesterol-lowering, Diabetes, Antihypertensive, Antidiarrhea, Anti-hemorrhage, Flavoring, Blood Refining	Mashhad city, Northeastern Iran Urmia county, Northwest Iran	[5] Amiri and Joharchi 2013 [7] Bahmani et al. 2014
***181.***	*Ribes khorasanicum* Saghafi & Assadi	Grossulariaceae	Ghareh Ghat	Fruit	n.r.	Antihypertensive, Diabetes, Depurative	Mashhad city, Northeastern Iran	[5] Amiri and Joharchi 2013
***182.***	*Ricinus communis* L.	Euphorbiaceae	Karchak	Seed	n.r.	Purgative	Mashhad city, Northeastern Iran	[5] Amiri and Joharchi 2013
***183.***	*Rosa beggeriana* Schrenk	Rosaceae	Nastaran	Fruit	n.r.	Antihypertensive, Diuretic, Kidney Stone	Mashhad city, Northeastern Iran	[5] Amiri and Joharchi 2013
***184.***	*Rosa damascena* Mill.	Rosaceae	Gole Mohammadi	Flower	n.r.	Anti-hemorrhoid, Laxative, Calmative	Mashhad city, Northeastern Iran	[5] Amiri and Joharchi 2013
***185.***	*Rosa foetida* Hermam. *Rosa foetida* Herrm.	Rosaceae Rosaceae	n.r. Gole Zard	Petal Flower	Boiled n.r.	Antidiabetic Ovary Tonic, Emmenagogue	Urmia county, Northwest Iran Mashhad city, Northeastern Iran	[7] Bahmani et al. 2014 [5] Amiri and Joharchi 2013
***186.***	*Rubia tinctorum* L. *Rubia tinctorum* L.	Rubiaceae Rubiaceae	Ronas Ronnas	Root Root, fruit	n.r. Boiled	Strengthening of Hair, Hair Color Hair Loss, Hair Coloring	Mashhad city, Northeastern Iran Khiregah-e Jangali, Ghasemloo valley	[5] Amiri and Joharchi 2013 [9] Baharvand-Ahmadi et al. 2015
***187.***	*Rumex acetosella* L.	Polygonaceae	Sagh Torshak	Root	n.r.	Jaundice, Febrifuge	Mashhad city, Northeastern Iran	[5] Amiri and Joharchi 2013
***188.***	*Rumex sculantus* L.	Polygonaceae	n.r.	Fruit, leaf	Raw use, boiled	Blood Refining	Urmia county, Northwest Iran	[7] Bahmani et al. 2014
***189.***	*Ruta graveolens* L.	Rutaceae	Sodab	Aerial parts	n.r.	Abortion, Sedative, Emmenagogue	Mashhad city, Northeastern Iran	[5] Amiri and Joharchi 2013
***190.***	*Salix aegyptiaca* L.	Salicaceae	Bidmeshk	Flower	n.r.	Calmative, Cardiac Tonic, Painful Menstruation	Mashhad city, Northeastern Iran	[5] Amiri and Joharchi 2013
***191.***	*Salix alba* L.	Salicaceae	Bid	Leaves-Bark	n.r.	Menstrual Pains, Anodyne, Jaundice, Antitussive	Mashhad city, Northeastern Iran	[5] Amiri and Joharchi 2013
***192.***	*Salix excelsa* J.F. Gmel.	Salicaceae	Bidkhesht	Manna	n.r.	Febrifuge, Jaundice, Laxative	Mashhad city, Northeastern Iran	[5] Amiri and Joharchi 2013
***193.***	*Salvia leriifolia* Benth.	Lamiaceae	Noruzak	Aerial parts	n.r.	Diabetes, Period Regulator	Mashhad city, Northeastern Iran	[5] Amiri and Joharchi 2013
***194.***	*Salvia macrosiphon* Boiss.	Lamiaceae	Kenocheh	Seed	n.r.	Jaundice, Antitussive, Febrifuge, Gastric Ulcer, Pharyngitis, Laxative	Mashhad city, Northeastern Iran	[5] Amiri and Joharchi 2013
***195.***	*Salvia nemorosa* L.	Lamiaceae	n.r.	Flowering offshoot	Boiled	Antidiabetic	Urmia county, Northwest Iran	[7] Bahmani et al. 2014
***196.***	*Sanguisorba minor* Scop. *Sanguisorba minor* Scop.	Rosaceae Rosaceae	Tout-e roubahi n.r.	Fruit Fruit, leaf	Boiled and edible raw Raw use, boiled	Skin Wounds Disinfection Antidiabetic	Khiregah-e Jangali, Ghasemloo valley Urmia county, Northwest Iran	[9] Baharvand-Ahmadi et al. 2015 [7] Bahmani et al. 2014
***197.***	*Satureja hortensis* L.	Lamiaceae	Marzeh	Aerial parts	n.r.	Indigestion, Anthelmintic, Appetizer, Antacid, Antidiarrhea	Mashhad city, Northeastern Iran	[5] Amiri and Joharchi 2013
***198.***	*Satureja khozistanica*	Lamiaceae	Jataneh	Branch	Dried leaves poured on food	Stomach Ache	Lorestan province	[8] Delfan et al. 2015
***199.***	*Scrophularia striata* Boiss.	Scrophulariaceae	Mokhallaseh	Aerial parts	n.r.	Kidney Troubles, Antidiarrhea, Treatment of Colic, Carminative, Treatment of Joints Pain	Mashhad city, Northeastern Iran	[5] Amiri and Joharchi 2013
***200.***	*Securigera securidaca* (L.) Degen & Dorfl.	Fabaceae	Gandeh Talkheh	Seed	n.r.	Diabetes, Antihyperlipidemia	Mashhad city, Northeastern Iran	[5] Amiri and Joharchi 2013
***201.***	*Sesamum indicum* L.	Pedaliaceae	Konjed	Seed	n.r.	Blood Tonic, Hair Loss, Strengthening of Memory, Increase Sperm Count, Treatment of Skin’s Split, Laxative	Mashhad city, Northeastern Iran	[5] Amiri and Joharchi 2013
***202.***	*Silybum marianum* (L.) Gaertn.	Asteraceae	Khare Maryam	Seed	n.r.	Jaundice, Febrifuge, Antihepatitis, Liver Tonic	Mashhad city, Northeastern Iran	[5] Amiri and Joharchi 2013
***203.***	*Solanum americanum* Mill.	Solanaceae	Tajrizi	Fruit	n.r.	Treatment of Osteoarthritis, Mastitis, Expectorant, Hypnotic, Sedative, Treatment of Gastritis	Mashhad city, Northeastern Iran	[5] Amiri and Joharchi 2013
***204.***	*Sophora alopecuroides*	Fabaceae	n.r.	Inflorescence	Boiled	Antidiabetic	Urmia county, Northwest Iran	[7] Bahmani et al. 2014
***205.***	*Stachys lavandulifolia* Vahl	Lamiaceae	Chai Kuhi	Flower	n.r.	Nerve Tonic, Treatment of cold, Cardiac Tonic, Treatment of Colic	Mashhad city, Northeastern Iran	[5] Amiri and Joharchi 2013
***206.***	*Tanacetum parthenium* (L.) Sch. Bip.	Asteraceae	Gole babooneh	Flower	n.r.	Antitussive, Anticatarrhal, Hair Tonic, Treatment of Colic, Menstrual Pains	Mashhad city, Northeastern Iran	[5] Amiri and Joharchi 2013
***207.***	*Teucrium orientale* L.	Lamiaceae	n.r.	Leaf	Boiled	Antidiabetic	Urmia county, Northwest Iran	[7] Bahmani et al. 2014
***208.***	*Teucrium polium* *Teucrium polium* L. *Teucrium* *polium* L.	Lamiaceae Lamiaceae Lamiaceae	Maryam-nokhodi Kalpureh n.r.	Flower, Seed Aerial parts Flowering offshoot	Brew n.r. Boiled	Stomach Ache Antacid, Indigestion, Diabetes, Treatment of Colic, Antidiarrhea Antidiabetic	Lorestan province Mashhad city, Northeastern Iran Urmia county, Northwest Iran	[8] Delfan et al. 2015 [5] Amiri and Joharchi 2013 [7] Bahmani et al. 2014
***209.***	*Thalictrum sultanabadense* Stapf	Ranunculaceae	Parsiavashan	Aerial parts	n.r.	Antitussive, Febrifuge	Mashhad city, Northeastern Iran	[5] Amiri and Joharchi 2013
***210.***	*Thymus daenensis*	Lamiaceae	Azboue	Flower, leaf, branch	Decoction	Stomach Ache	Lorestan province	[8] Delfan et al. 2015
***211.***	*Thymus kotschyanus*	Lamiaceae	Azboue	Flower, leaf, branch	Decoction	Stomach Ache	Lorestan province	[8] Delfan et al. 2015
***212.***	*Thymus pubescens*	Lamiaceae	Azboue	Flower, leaf, branch	Decoction	Stomach Ache	Lorestan province	[8] Delfan et al. 2015
***213.***	*Thymus fallax*	Lamiaceae	Azboue	Flower, leaf, branch	Decoction	Stomach Ache	Lorestan province	[8] Delfan et al. 2015
***214.***	*Thymus. eriocalyx*	Lamiaceae	Azboue	Flower, leaf, branch	Decoction	Stomach Ache	Lorestan province	[8] Delfan et al. 2015
***215.***	*Tilia cordata* Mill.	Malvaceae	Zirfun	Leaves - Fruit	n.r.	Nerve Tonic, Sudorific, Diuretic, Calmative	Mashhad city, Northeastern Iran	[5] Amiri and Joharchi 2013
***216.***	*Trachyspermum ammi* (L.) Sprague	Apiaceae	Zenyan (Khordaneh)	Fruit	n.r.	Carminative, Anthelmintic, Antidiarrhea, Treatment of Colic, Antacid, Galactogogue	Mashhad city, Northeastern Iran	[5] Amiri and Joharchi 2013
***217.***	*Tragapogon caricifolius*	Compositae	Sheng	Flower	Brew, raw, dried	Stomach Ache	Lorestan province	[8] Delfan et al. 2015
***218***	*Tribulus terrestris* L.	Zygophyllaceae	Kharkhasak	Aerial parts	n.r.	Diuretic, Kidney Stone, Tonic, Treatment of Prostate, Hypertrophy, Anthelmintic, Jaundice, Treatment of Flooding, Treatment of Dysuria, Urinary Antiseptic	Mashhad city, Northeastern Iran	[5] Amiri and Joharchi 2013
***219.***	*Trichodesma incanum* (Bunge) A. DC.	Boraginaceae	Alaf-e-simkesh	Aerial parts	n.r.	Treatment of Bone Fracture	Mashhad city, Northeastern Iran	[5] Amiri and Joharchi 2013
***220.***	*Trifolium pratense* L.	Fabaceae	n.r.	Flowering offshoot	Boiled	Antidiabetic	Urmia county, Northwest Iran	[7] Bahmani et al. 2014
***221.***	*Trifolium purpureum* Loisel.	Fabaceae	n.r.	Flowering offshoot	Boiled	Antidiabetic	Urmia county, Northwest Iran	[7] Bahmani et al. 2014
***222.***	*Trigonella foenum- graecum* L.	Fabaceae	Shanbalileh (Holbeh)	Seed	n.r.	Diabetes, Bronchitis, Osteomalacia, Antihyperlipidemia, Tonic, Treatment of Anemia	Mashhad city, Northeastern Iran	[5] Amiri and Joharchi 2013
***223.***	*Tripleurospermum disciforme* (C. A. Mey.) Sch.Bip.	Asteraceae	Gole babooneh	Flower	n.r.	Treatment of Cough, Febrifuge	Mashhad city, Northeastern Iran	[5] Amiri and Joharchi 2013
**224.**	Tussilago farfara L.	Asteraceae	Pa Khari	Aerial parts	n.r.	Expectorant, Antitussive, Mouth Wounds, Treatment of Furuncles	Mashhad city, Northeastern Iran	[5] Amiri and Joharchi 2013
***225.***	*Urtica dioica* L. *Urtica. dioica* L.	Urticaceae Urticaceae	Gazaneh n.r.	Whole plant Seed, aeration organ	n.r. Boiled	Hypoglycemic, Enlarged Prostate, Anemia, Anti-inflammatory, Digestive Antidiabetic	Mashhad city, Northeastern Iran Urmia county, Northwest Iran	[5] Amiri and Joharchi 2013 [7] Bahmani et al. 2014
***226.***	*Urtica pilulifera* L.	Urticaceae	Anjareh	Seed	n.r.	Laxative, Treatment of Cough	Mashhad city, Northeastern Iran	[5] Amiri and Joharchi 2013
***227.***	*Vaccaria oxyodonta* Boiss.	Caryophyllaceae	Sabounak-e dane-ye zard	Flower	Boiled	Skin Allergy and Constipation	Khiregah-e Jangali, Ghasemloo valley	[9] Baharvand-Ahmadi et al. 2015
***228.***	*Vaccinium arctostaphylos* L.	Ericaceae	Ghareh Ghat	Fruit	n.r.	Diabetes, Depurative, Antihypertensive, Calmative	Mashhad city, Northeastern Iran	[5] Amiri and Joharchi 2013
***229.***	*Verbascum agrimonifolium*	Scropholariacae	Gol-e mahour	Leaves, flower	Boiled	Wound Microbial Infection	Khiregah-e Jangali, Ghasemloo valley	[9] Baharvand-Ahmadi et al. 2015
***230.***	*Verbascum cheiranthifolium* Boiss.	Scrophulariaceae	Dome Gav	Aerial parts	n.r.	Dyspepsia, Antidiarrhea, Expectorant, Antiacid, Stomach Tonic	Mashhad city, Northeastern Iran	[5] Amiri and Joharchi 2013
***231.***	*Verbascum macrocarpum* Boiss.	Scropholariacae	Gol-e mahour	Leaves, flower	Boiled	Nails Fungal Infection	Khiregah-e Jangali, Ghasemloo valley	[9] Baharvand-Ahmadi et al. 2015
***232.***	*Verbascum speciosum* Schord.	Scropholariacae	Gol-e mahour	Leaves, flower	Paste	Wound Microbial Infection	Khiregah-e Jangali, Ghasemloo valley	[9] Baharvand-Ahmadi et al. 2015
***234.***	*Verbena officinalis* L.	Verbenaceae	Shahpasand	Aerial parts	n.r.	Appetizer, Indigestion	Mashhad city, Northeastern Iran	[5] Amiri and Joharchi 2013
***235.***	*Viola odorata* L.	Violaceae	Banafsheh	Flower	n.r.	Eczema, Febrifuge, Anti-allergic, Blood Cleansing, Jaundice, Treatment of Cold, Expectorant	Mashhad city, Northeastern Iran	[5] Amiri and Joharchi 2013
***236.***	*Viola tricolor*	Umbelliferae	Gole- benoushe	Flower branch	Decoction	Stomach Ache	Lorestan province	[8] Delfan et al. 2015
***237.***	*Vitex negundo* L.	Lamiaceae	Felfel Kuhi	Fruit	n.r.	Menstrual Regulator, Obesity, Treatment of Sinusitis	Mashhad city, Northeastern Iran	[5] Amiri and Joharchi 2013
***238.***	*Zataria multiflora* Boiss.	Lamiaceae	Avishan Shirazi	Aerial parts	n.r.	Treatment of Sinusitis, Menstrual Pains, Dysmenorrheal, Anthelmintic, Antacid, Treatment of Colic, Anti-asthmatic, Dyspnoea, Arthrodynia, Carminative	Mashhad city, Northeastern Iran	[5] Amiri and Joharchi 2013
***239.***	*Zataria multiflora*	Lamiceae	Avishan	Leaf	Herbal tea/decoction	Antiparasitic	Shiraz, Fars province	[6] Bahmani et al. 2016
***240.***	*Zea mays* L.	Poaceae	Kakole Zorat	Style	n.r.	Obesity, Anti-inflammatory, Anti-lithiasis, Kidney Disorders, Prostate Disorders, Diuretic	Mashhad city, Northeastern Iran	[5] Amiri and Joharchi 2013
***241.***	*Ziziphora clinopodioides* Lam.	Lamiaceae	Avishan kuhi	Aerial parts	n.r.	Kidney Pain, Antacid, Carminative, Treatment of Colic, Anthelmintic, Antitussive, Antidiarrhea, Digestive	Mashhad city, Northeastern Iran	[5] Amiri and Joharchi 2013
***242.***	*Ziziphora teniuor* L.	Lamiaceae	Kakuti	Aerial parts	n.r.	Digestive, Treatment of Colic, Calefacient, Antacid, Antiseptic	Mashhad city, Northeastern Iran	[5] Amiri and Joharchi 2013
***243.***	*Ziziphus jujuba* Miller	Rhamnaceae	Annab	Fruit	n.r.	Depurative, Febrifuge, Laxative, Jaundice, Antitussive, Treatment of Thirst	Mashhad city, Northeastern Iran	[5] Amiri and Joharchi 2013
***244.***	*Ziziphus spina-christi* *Ziziphus spina-christi* (L.) Willd.	Rhamnaceae Rhamnaceae	Konar Sedr	Flower, leaf Leaves	Decoction n.r.	Stomach Ache Eczema, Hair Tonic, Antifungal, Antipruritic, Washing	Lorestan province Mashhad city, Northeastern Iran	[8] Delfan et al. 2015 [5] Amiri and Joharchi 2013
***245.***	*Ziziphus nummularia*	Rhamnaceae	Melim	Leaf, root	Decoction	Peptic Ulcer	Lorestan province	[8] Delfan et al. 2015
***246.***	*Zosima orientalis* Hoffm.	Apiaceae	Angedane roomi	Fruit	n.r.	Nerve Diseases, Indigestion	Mashhad city, Northeastern Iran	[5] Amiri and Joharchi 2013

**Table 2:** Biological activities of plants collected in the Iranian territory. Scientific name, family, type of extract, part of the plant used, Authors. (N.r. = not reported).

	Family	Plant Extract	Plant Part (s) Used	Author (s)
**Antibacterial Activity**				
***Achillea millefolium***	Asteraceae	Methanolic extract	Aerial parts	[10] Lotfipour et al. 2008
***Alhagi maurorum* Medik.**	Leguminosae	Methanolic extract (Lyophilized)	Leaves	[11] Bonjar et al. 2004
***Beta vulgaris***	Amaranthaceae	Ethanolic extract	Aerial parts	[12] Koochak et al. 2010
***Cuminum cyminum* L.**	Apiaceae	Methanolic extract (Lyophilized)	Leaves	[11] Bonjar et al. 2004
***Dorema ammoniacum***	Apiaceae	Methanolic extract	Seeds	[13] Abedini et al. 2014
***Echinophora orientalis***	Apiaceae	Aqueous extract	Leaves	[14] Sepahi et al. 2014
***Etchium italicum***	Boraginaceae	Methanolic extract	Aerial parts	[10] Lotfipour et al. 2008
***Ferula assa-foetida***	Apiaceae	Methanolic extract	Seeds	[13] Abedini et al. 2014
***Ferula foetida* Regel**	Apiaceae	Methanolic extract	Roots	[15] Chitsazian-Yazdi et al. 2015
***Ferula gummosa***	Apiaceae	Aqueous extract	Leaves	[14] Sepahi et al. 2014
***Ferulago contracta***	Apiaceae	Methanolic extract	Seeds	[13] Abedini et al. 2014
***Lawsonia inermis* L.**	Lythraceae	Methanolic extract (Lyophilized)	Leaves	[11] Bonjar et al. 2004
***Malva sylvestris* L.**	Malvaceae	Methanolic extract	Flowers	[16] Razavi et al. 2011
***Nasturtium microphyllum***	Brassicaceae	Aqueous extract	Leaves	[14] Sepahi et al. 2014
***Nymphaea alba* L.**	Nymphaeaceae	Methanolic extract (Lyophilized)	Leaves	[11] Bonjar et al. 2004
***Perovskia abrotanoides***	Lamiaceae	Methanolic extract	Aerial parts	[13] Abedini et al. 2014
***Polygonum patulum*** **M. Bieb.**	Polygonaceae	Ethanolic extract	Aerial parts	[12] Koochak et al. 2010
***Rheum ribes* L.**	Polygonaceae	Methanolic extract (Lyophilized)	Leaves	[11] Bonjar et al. 2004
***Rhus coriaria* L.**	Anacardiaceae	Methanolic extract (Lyophilized)	Leaves	[11] Bonjar et al. 2004
***Rumex obtusifolius***	Polygonaceae	Ethanolic extract	Aerial parts	[12] Koochak et al. 2010
***Salvia sahendica***	Lamiaceae	Methanolic extract	Aerial parts	[10] Lotfipour et al. 2008
***Satureja bachtiarica***	Lamiaceae	Hydro-distillation and ethanolic extract	Leaves and flowers	[17] Pirbalouti et al. 2010
***Thalictrum minus***	Ranunculaceae	Methanolic extract	Aerial parts	[10] Lotfipour et al. 2008
***Thymus daenensis***	Lamiaceae	Hydro-distillation and ethanolic extract	Leaves and flowers	[17] Pirbalouti et al. 2010
***Trachyspermum ammi* L.**	Apiaceae	Methanolic extract (Lyophilized)	Leaves	[11] Bonjar et al. 2004
***Trachyspermum copticum***	Apiaceae	Aqueous extract, Methanol/petroleum benzene/diethyl ether extract	Aerial parts	[18] Nariman et al. 2004
***Trigonella foenum-graecum* L.**	Leguminosae	Methanolic extract (Lyophilized)	Leaves	[11] Bonjar et al. 2004
***Verbascum Thapsus***	Scrophulariaceae	Aqueous extract	Leaves	[14] Sepahi et al. 2014
***Xanthium brasilicum***	Compositae	Aqueous extract, Methanol/petroleum benzene/diethyl ether extract	Aerial parts	[18] Nariman et al. 2004
**Antifungal Activity**				
***Satureja bachtiarica***	Lamiaceae	Hydro-distillation	Leaves	[19] Pirbalouti et al. 2009
***Scrophularia striata***	Scrophulariaceae	Infusion	Leaves and stems	[19] Pirbalouti et al. 2009
***Thymus daenensis***	Lamiaceae	Hydro-distillation	Leaves	[19] Pirbalouti et al. 2009
***Trachyspermum ammi***	Apiaceae	Hydro-distillation	Fruits	[19] Pirbalouti et al. 2009
***Zhumeria majdae***	Lamiaceae	Hydro-distillation	Aerial parts	[20] Imani et al. 2015
***Ziziphus spinachristi***	Rhamnaceae	Infusion	Fruits	[19] Pirbalouti et al. 2009
**Antimalarial Activity**				
***Citrullus colocynthis***	Cucurbitaceae	Methanolic extract	Fruits	[21] Feiz Haddad et al. 2017
***Physalis alkekengi***	Solanaceae	Methanolic extract	Leaves and fruits	[21] Feiz Haddad et al. 2017
***Scrophularia frigida***	Scrophulariaceae	Dichloromethane extract	Aerial parts	[22] Afshar et al. 2018
***Solanum nigrum***	Solanaceae	Methanolic extract	Fruits	[21] Feiz Haddad et al. 2017
**Antioxidant Activity**				
***Convolvulus persicus***	Convolvulaceae	Methanol extract	Roots	[23] Dehghan et al. 2016
***Heracleum persicum***	Apiaceae	n-Hexane extract (subsequently fractionated)	Roots	[24] Dehghan et al. 2017
***Hyssopus angustifolius***	Lamiaceae	Ethyl acetate extracts	Stems, Leaves, Owes	[25] Alinezhad et al. 2012
***Hyssopus officinalis* L.**	Lamiaceae	Ethyl acetate and n-butanol extracts	Aerial parts	[26] Fathiazad et al. 2011
***Mellilotus officinalis***	Leguminosae	Methanolic extract	Whole plant	[27] Pourmorad et al. 2006
***Primula heterochroma***	Primulaceae	Methanolic extract Ethyl acetate extract Methanolic extract	Leaves Roots	[23] Dehghan et al. 2016
***Pyrus boissieriana***	Rosacee	Methanolic extract	Leaves and steams	[23] Dehghan et al.2016
***Quercus infectoria***	Fagaceae	Methanolic extract	Galls	[28] Khazaeli et al. 2009
***Salix aegyptiaca L.***	Salicaceae	Methanolic extract	Male inflorescences	[29] Sonboli et al. 2010
***Stachys inflata***	Lamiaceae	Methanolic extract polar and non-polar fractions	Aerial parts	[30] Ebrahimabadi et al. 2010
***Terminalia chebula***	Combretaceae	Methanolic extract	Fruits	[28] Khazaeli et al. 2009
***Tetrataenium lasiopetalum***	Apiaceae	Hydro-alcoholic extract	Laminas, Stems, Petioles, Fruits, Peduncle, Flowers	[31] Dehshiri et al. 2013
**Anticancer/Cytotoxic Activity**				
***Anthemis mirheydari***	Compositae	Dichloromethane extract	Whole plant	[32] Jassbi et al. 2016
***Euphorbia szovitsii* Fisch. & C.A. Mey.**	Euphorbiaceae	Hydro-alcoholic extract	Aerial parts	[33] Asadi-Samani et al. 2018
***Ferula foetida* Regel**	Apiaceae	Methanolic extract	Roots	[15] Chitsazian-Yazdi et al. 2015
***Ferula szowitsiana***	Apiaceae	Methanolic extract (fractionated)	Roots	[34] Sahranavard et al. 2009
***Hypericum scabrum***	Hypericaceae	Methanolic extract (fractionated)	Leaves	[35] Hamzeloo-Moghadam et al. 2015
***Malva sylvestris* L.**	Malvaceae	Methanolic extract	Flowers and leaves	[16] Razavi et al. 2011
***Medicago sativa***	Leguminosae	Hydro-alcoholic extract	Aerial parts	[33] Asadi-Samani et al. 2018
***Mentha lonigfolia***	Lamiaceae	Methanolic extract	Aerial parts	[36] Esmaeilbeig et al. 2015
***Satureja bachtiarica***	Lamiaceae	Methanolic extract	Aerial parts	[36] Esmaeilbeig et al. 2015
***Satureja hortensis***	Lamiaceae	Methanolic extract	Aerial parts	[36] Esmaeilbeig et al. 2015
***Thymus daenensis***	Lamiaceae	Methanolic extract	Aerial parts	[36] Esmaeilbeig et al. 2015
***Thymus vulgaris***	Lamiaceae	Methanolic extract	Aerial parts	[36] Esmaeilbeig et al. 2015
***Urtica dioica***	Urticaceae	Hydro-alcoholic extract	Aerial parts	[33] Asadi-Samani et al. 2018
**Antidiabetic Activity**				
***Heracleum persicum***	Apiaceae	n-hexane extract n-hexane extract (subsequently fractionated)	Aerial parts, roots Roots	[23] Dehghan et al. 2016 and [24] Dehghan et al. 2017
***Parrotia persica***	Hamamelidaceae	Ethyl acetate and methanolic extract	Leaves	[23] Dehghan et al.2016
***Primula heterochroma***	Primulaceae	Methanolic and ethyl acetate extract	Leaves and roots	[23] Dehghan et al. 2016
***Pyrus boissieriana***	Rosacee	Methanolic, n-hexane, Ethyl acetate extract	Leaves and stems	[23] Dehghan et al.2016
***Salvia officinalis* L**	Lamiaceae	Hydro-alcoholic extract	Leaves	[37] Hasanein et al. 2016
***Smilax excelsa***	Smilacaceae	Ethyl acetate and n-hexane extract	Stems and leaves	[23] Dehghan et al.2016
**Iron Chelating Activity**				
***Epilobium hirsutum***	Onagraceae	n.r.	Leaves	[38] Ebrahimzadeh et al. 2008
***Feijoa sellowiana***	Myrtaceae	Infusion and methanolic extract	Fruits and leaves	[38] Ebrahimzadeh et al. 2008
***Melilotus arvensis***	*Fabaceae*	n.r.	Leaves	[38] Ebrahimzadeh et al. 2008
***Pistacia lentiscus***	Anacardiaceae	n.r.	Gum	[38] Ebrahimzadeh et al. 2008
**Anti-Platelet Aggregation Activity**				
***Allium atroviolaceum***	Amaryllidaceae	Hydro-distillation	Aerial parts	[39] Lorigooini et al. 2014
**Inhibition of Mushroom Tyrosinase**				
***Quercus infectoria***	Fagaceae	Methanolic extract	Galls	[28] Khazaeli et al. 2009
***Terminalia chebula***	Combretaceae	Methanolic extract	Fruits	[28] Khazaeli et al. 2009
**Acetylcholinesterase-Inhibitory Activity**				
***Brassica nigra***	Brassicaceae	Aqueous-methanolic extract	Seeds	[40] Jazayeri et al. 2014
***Camellia sinensis***	Theaceae	Aqueous-methanolic extract	Leaves	[40] Jazayeri et al. 2014
***Citrus aurantifolia***	Rutaceae	Aqueous-methanolic extract	Fruits	[40] Jazayeri et al. 2014
***Peganum harmala* L.**	Nitrariaceae	Methanolic extract, Dichloromethane extract	Seeds	[41] Adhami et al. 2011
***Prangos ferulacea***	Apiaceae	n-hexane extract	Aerial parts	[42] Abbas-Mohammadi et al. 2018
***Rosa damascena***	Rosaceae	Aqueous-methanolic extract	Flowers	[40] Jazayeri et al. 2014
***Zizyphus vulgaris***	Rhamnaceae	Aqueous-methanolic extract	Fruits	[40] Jazayeri et al. 2014
**Antihyperlipidemic and Antihypertensive Activities**				
***Achillea wilhelmsii* C. Koch**	Compositae	Hydro-alcoholic extract	Aerial parts	[43] Asgary et al. 2000
**Gastric Antiulcerogenic Activity**				
***Portulaca oleracea* L.**	Portulacaceae	Aqueous extract Ethanolic extract	Leaves	[44] Karimi et al. 2004
**Anti-Dyspepsia Activity**				
***Mentha pulegium* L.**	Lamiaceae	Hydro-alcoholic extract	Leaves	[45] Khonche et al. 2017
**Inhibitory Effect on Gastric Acid Output**				
***Achillea wilhelmsii***	Compositae	Aqueous-ethanolic extract	Aerial parts	[46] Niazmand et al. 2010
**Anti-Colitic Activity**				
***Rosmarinus officinalis***	Lamiaceae	Hydro-alcoholic extract and hydro-distillation (EO)	Leaves	[47] Minaiyan et al. 2011

**Table 3:** Comparison between traditional uses and tested biological activities of Iranian plants. (N.r. = not reported).

Plant Name Family	Traditional Uses Part of the Plant (When Reported) Type of Extract (When Reported) Authors	Biological Activities Part of the Plant Type of Extract Authors
***Achillea millefolium* L.** **Asteraceae**	Antidiabetic Inflorescence Boiled, Steamed [7] Bahmani et al. *2014* Antiparasitic Aerial parts Herbal tea/decoction [6] Bahmani et al. 2016	Antibacterial activity Aerial parts Methanolic extract [10] Lotfipour et al. 2008
***Alhagi maurorum*** **Fabaceae**	Appetite suppressant, Diuretic, Jaundice, Febrifuge Aerial parts, Manna [5] Amiri and Joharchi 2013	Antibacterial activity Leaves Methanolic extract (Lyophilized) [11] Bonjar et al. 2004
***Brassica nigra*(L.)** **Brassicaceae**	Laxative Seeds [5] Amiri and Joharchi 2013	Acetylcholinesterase-inhibitory activity Seeds Aqueous-methanolic extract [40] Jazayeri et al. 2014
***Camellia sinensis* (L.)** **Theaceae**	Obesity, Anticancer, Antihypertensive, Hepatitis, Antihyperlipidemia Leaves [5] Amiri and Joharchi 2013	Acetylcholinesterase-inhibitory activity Leaves Aqueous-methanolic extract [40] Jazayeri et al. 2014
***Citrulluscolocynthis* (L.)** **Cucurbitaceae**	Antidiabetic Fruit Boiled [7] Bahmani et al. *2014* Purgative, Anodyne, Hypoglycemic Fruit- Seed [5] Amiri and Joharchi 2013	Antimalarial activity Fruits Methanolic extract [21] Feiz Haddad et al. 2017
***Citrus aurantiifolia*** **Rutaceae**	Antihypertensive, Calmative Fruit [5] Amiri and Joharchi 2013	Acetylcholinesterase-inhibitory activity Fruits Aqueous-methanolic extract [40] Jazayeri et al. 2014
***Cuminum cyminum* L.** **Apiaceae**	Treatment of colic, Galactogogue, Obesity, Digestive, Flavoring, Antiseptic Fruit [5] Amiri and Joharchi 2013	Antibacterial activity Leaves Methanolic extract (Lyophilized) [11] Bonjar et al. 2004
***Dorema ammoniacum*** **Apiaceae**	Cystitis, Digestive, Treatment of colic, Treatment of furuncles, Expectorant, Anthelmintic, Emmenagogue, Anticovulsion Gum- Root [5] Amiri and Joharchi 2013	Antibacterial activity Seeds Methanolic extract [13] Abedini et al. 2014
***Ferula assa-foetida L*** **Umbelliferae**	Antiparasitic Leaf Herbal tea/decoction [6] Bahmani et al. 2016	Antibacterial activity Seeds Methanolic extract [13] Abedini et al. 2014
***Ferula foetida*** **Apiaceae**	Anthelmintic, Treatment of colic, Emmenagogue Gum [5] Amiri and Joharchi 2013	Antibacterial activity Roots Methanolic extract [15] Chitsazian-Yazdi et al. 2015 Cytotoxic activity Roots Methanolic extract [15] Chitsazian-Yazdi et al. 2015
***Ferula gummosa*** **Apiaceae**	Anthelmintic, Anticatarrhal, Anti-allergic, Dyspepsia, Appetizer, Emmenagogue Gum- Root [5] Amiri and Joharchi 2013	Antibacterial activity Leaves Aqueous extract [14] Sepahi et al. 2014
***Heracleum persicum*** **Apiaceae**	Stomach ache Leaf, Flower Decoction [8] Delfan et al. 2015 Treatment of hiccup, Appetizer, Flavoring, Carminative, Anthelmintic, Stomach tonic fruit [5] Amiri and Joharchi 2013	Antioxidant activity Roots n-Hexane extract (subsequently fractionated) [24] Dehghan et al. 2017 Antidiabetic activity Aerial parts, Roots n-hexane extract Roots n-Hexane extract (subsequently fractionated) [23] Dehghan et al.2016 and [24] Dehghan et al. 2017
***Hypericum scabrum* L.** **Hypericaceae**	Antimigraine, Gastric ulcer, Anti-hemorrhage, Urinary incontinence, Treatment of headache Flower [5] Amiri and Joharchi 2013	Cytotoxic activity and apoptosis induction activity Leaves Methanolic extract (fractionated) [35] Hamzeloo-Moghadam et al. 2015
***Lawsonia inermis* L.** **Lythraceae**	Hair color, Treatment of headache, Hair tonic, Washing, Antifungal, Antiseptic Leaves [5] Amiri and Joharchi 2013	Antibacterial activity Leaves Methanolic extract (Lyophilized) [11] Bonjar et al. 2004
***Malva sylvestris* L.** **Malvaceae**	Pharyngitis, Furuncles, Aphthous ulcers, Febrifuge, Antitussive, Jaundice, Laxative, Gastric ulcer, Treatment of wounds Flower– Fruit [5] Amiri and Joharchi 2013	Antibacterial activity Flowers Methanolic extract [16] Razavi et al. 2011 Cytotoxic activity Flowers and leaves Methanolic extract [16] Razavi et al. 2011
***Medicago sativa* L.** **Fabaceae**	Appetizer, Tonic, Osteomalacia, Anti-hemorrhage Aerial parts [5] Amiri and Joharchi 2013	Antiproliferative on DU-145 cell line Aerial parts Hydro-alcoholic extract [33] Asadi-Samani et al. 2018
***Mentha longifolia*** **Lamiaceae**	Herpes, Anthelmintic, Antacid, Carminative, Antidiarrhea, Digestive Aerial parts [5] Amiri and Joharchi 2013	Anticancer activity Aerial parts Methanolic extract [36] Esmaeilbeig et al. 2015
***Nymphaea alba* L.** **Nymphaeaceae**	Expectorant, Hypnotic, Antitussive, Calmative Flower [5] Amiri and Joharchi 2013	Antibacterial activity Leaves Methanolic extract (Lyophilized) [11] Bonjar et al. 2004
***Peganum harmala* L.** **Nitrariaceae**	Diabetes, Antiseptic, Hypnotic, Treatment of rheumatism and sciatica disorders, Anthelmintic, Emmenagogue Seed [5] Amiri and Joharchi 2013	Acetylcholinesterase-inhibitory activity Seeds Methanolic extract and dichloromethane extract [41] Adhami et al. 2011
***Perovskia abrotanoides* Kar.** **Lamiaceae**	Treatment of sinusitis, Treatment of toothache, Antitussive, Nerve tonic, Carminative, Sedative, Antiseptic, Anthelmintic, Treatment of colic Aerial parts [5] Amiri and Joharchi 2013	Antibacterial activity Aerial parts Methanolic extract [13] Abedini et al. 2014
***Physalis alkekengi* L.** **Solanaceae**	Emmenagogue, Treatment of kidney stones, Blood cleansing Fruit [5] Amiri and Joharchi 2013	Antimalarial activity Leaves and fruits Methanolic extract [21] Feiz Haddad *et al.2017*
***Portulaca oleracea* L.** **Portulacaceae**	Antitussive, Febrifuge, Anti-thirst, Food digestion, Depurative, Diuretic, Anti-hemorrhoids Seed- Leaves [5] Amiri and Joharchi 2013	Gastric antiulcerogenic activity Leaves Aqueous extract and Ethanolic extract [44] Karimi et al. 2004
***Quercus infectoria* Oliv.** **Fagaceae**	Nosebleed, Anti-hemorrhage, Uterus ailments, Mouth wounds, Anti-hemorrhoids Insect gull [5] Amiri and Joharchi 2013	Radical scavenging activity Inhibition of mushroom tyrosinase Galls Methanolic extract [28] Khazaeli et al. 2009
***Rheum ribes* L.** **Polygonaceae**	Jaundice, Urinary antiseptic, Diuretic, Depurative, Liver tonic, Antiseptic, Hair tonic Fruit- Petiole [5] Amiri and Joharchi 2013	Antibacterial activity Leaves Methanolic extract (Lyophilized) [11] Bonjar et al. 2004
***Rhus coriaria* L.** **Anacardiaceae**	Jaundice, Cholesterol-lowering, Diabetes, Antihypertensive, Antidiarrhea, Anti-hemorrhage, Flavoring Fruit [5] Amiri and Joharchi 2013 Blood refining Fruit, Leaf, Resin Boiled [7] Bahmani et al. *2014*	Antibacterial activity Leaves Methanolic extract (Lyophilized) [11] Bonjar et al. 2004
***Rosa damascena* Mill.** **Rosaceae**	Anti-hemorrhoid, Laxative, Calmative Flower [5] Amiri and Joharchi 2013	Acetylcholinesterase-inhibitory activity Flowers Aqueous extract and methanolic extract [40] Jazayeri et al. 2014
***Salix aegyptiaca* L.** **Salicaceae**	Calmative, Cardiac tonic, Painful menstruation Flower [5] Amiri and Joharchi 2013	Antioxidant activity Male inflorescences Methanolic extract [29] Sonboli et al. 2010
***Satureja hortensis* L.** **Lamiaceae**	Indigestion, Anthelmintic, Appetizer, Antacid, Antidiarrhea Aerial parts [5] Amiri and Joharchi 2013	Anticancer activity Aerial parts Methanolic extract [36] Esmaeilbeig et al. 2015
***Scrophularia striata* Scrophulariaceae**	Kidney troubles, Antidiarrhea, Treatment of colic, Carminative, Treatment of joints pain Aerial parts [5] Amiri and Joharchi 2013	Anti-*Candida* activity Leaves and stems Infusion [19] Pirbalouti et al. 2009
***Thymus daenensis*** **Lamiaceae**	Stomach ache Flower, Leaf, Branch Decoction [8] Delfan et al. 2015	Antibacterial activity Leaves and flowers Hydro-distillation and ethanolic extract [17] Pirbalouti et al. 2010 Anti-*Candida* activity Leaves Hydro-distillation [19] Pirbalouti et al. 2009 Anticancer activity Aerial parts Methanolic extract [36] Esmaeilbeig et al. 2015
***Trachyspermum ammi* L.** **Apiaceae**	Carminative, Anthelmintic, Antidiarrhea, Treatment of colic, Antacid, Galactogogue Fruit [5] Amiri and Joharchi 2013	Antibacterial activity Leaves Methanolic extract (Lyophilized) [11] Bonjar et al. 2004 Anti-*Candida* activity Fruits Hydro-distillation [19] Pirbalouti et al. 2009
***Trigonella foenum- graecum* L.** **Fabaceae**	Diabetes, Bronchitis, Osteomalacia, Antihyperlipidemia, Tonic, Treatment of anemia Seed [5] Amiri and Joharchi 2013	Antibacterial activity Leaves Methanolic extract (Lyophilized) [11] Bonjar et al. 2004
***Urtica dioica* L.** **Urticaceae**	Hypoglycemic, Enlarged prostate, Anemia, Anti-inflammatory, Digestive Whole plant [5] Amiri and Joharchi 2013 Antidiabetic Seed, Aeration organ Boiled [7] Bahmani et al. *2014*	Antiproliferative on DU-145 cell line Aerial parts Hydro-alcoholic extract [33] Asadi-Samani et al. 2018
***Ziziphus spina-christi*** **Rhamnaceae**	Stomach ache Flower, Leaf Decoction [8] Delfan et al. 2015 Eczema, Hair tonic, Antifungal, Antipruritic, Washing Leaves [5] Amiri and Joharchi 2013	Anti-*Candida* activity Fruits Infusion [19] Pirbalouti et al. 2009
